# An 80 kyr-long continuous speleothem record from Dim Cave, SW Turkey with paleoclimatic implications for the Eastern Mediterranean

**DOI:** 10.1038/srep13560

**Published:** 2015-09-04

**Authors:** Ezgi Ünal-İmer, James Shulmeister, Jian-Xin Zhao, I. Tonguç Uysal, Yue-Xing Feng, Ai Duc Nguyen, Galip Yüce

**Affiliations:** 1 Climate Research Group, School of Geography, Planning & Environmental Management, The University of Queensland, Brisbane, QLD 4072, Australia; 2 Queensland Geothermal Energy Centre of Excellence, The University of Queensland, Brisbane QLD 4072, Australia; 3 School of Earth Sciences, The University of Queensland, Brisbane QLD 4072, Australia; 4 Department of Geological Engineering, Hacettepe University, TR 06800, Turkey

## Abstract

Speleothem-based stable isotope records are valuable in sub-humid and semi-arid settings where many other terrestrial climate proxies are fragmentary. The Eastern Mediterranean is one such region. Here we present an 80-kyr-long precisely-dated (by U-series) and high-resolution oxygen (δ^18^O) and carbon (δ^13^C) records from Dim Cave (~36°N) in SW Turkey. The glacial-interglacial δ^18^O variations in the Dim Cave speleothem are best explained in terms of changes in the trajectories of winter westerly air masses. These are along a northerly (European) track (isotopically less depleted) during the early last glaciation but are gradually depressed southward closer to the modern westerly track along the North African coast (more depleted) after c.50 kyr and remain in the southern track through the Last Glacial Maximum. The southward displacement of the westerly track reflects growth of the Fennoscandian ice sheet and its impact on westerly wind fields. Changes in δ^13^C are interpreted as reflecting soil organic matter composition and/or thickness. δ^13^C values are significantly more negative in interglacials reflecting active carbonic acid production in the soil and less negative in glacial times reflecting carbonate rock values. Several Heinrich events are recorded in the Dim record indicating intensification of westerly flow across this part of the EM.

Understanding the climate history of the Eastern Mediterranean (EM) region is of global significance because it lies on the boundary between two major climate regimes, the northern hemisphere westerlies and the sub-tropical anticyclonic belt, and the region has the potential to unlock long-term changes in the track and strength of the westerlies and the intensity of the summer anti-cyclone. To date, high resolution (up to 250 kyrs^1^,^2^,^3^,^4^ long) speleothem records have come from Soreq (~31°N) and Peqiin (~33°N) caves in Israel. These records are derived from the semi-arid southernmost part of the EM. They may be subject to more tropical influences and have, in the main, being exploited for the interglacial portion of their records. There is a paucity of records from further north. The longest (50-kyr-long) and best dated terrestrial climate record from the northern EM comes from Sofular Cave (41°N) situated in the northwest Black Sea region of Turkey (Fig. 1a). This region has a climate that is modified by the Black Sea and northerly air flows^5^,^6^,^7^ unlike the rest of the EM. In contrast, coastal western Turkey has a true Mediterranean climate and is an ideal location to investigate long-term changes in the behaviour of the Northern Hemisphere (NH) winter westerlies and the summer anti-cyclone. Here we present new δ^18^O and δ^13^C profiles for three U-series-dated stalagmites (Dim-E2, Dim-E3, and Dim-E4) from Dim Cave in southwestern Turkey (Fig. 1a,b). The main focus of the paper is on stalagmite Dim-E3 (~90–13 kyr) but Dim-E2 and Dim-E4 are being reported also so as to provide additional detail during the last deglaciation between 15–10 kyr (Table S1 online).

## Regional setting and climate of Dim Cave

**Figure 1:**
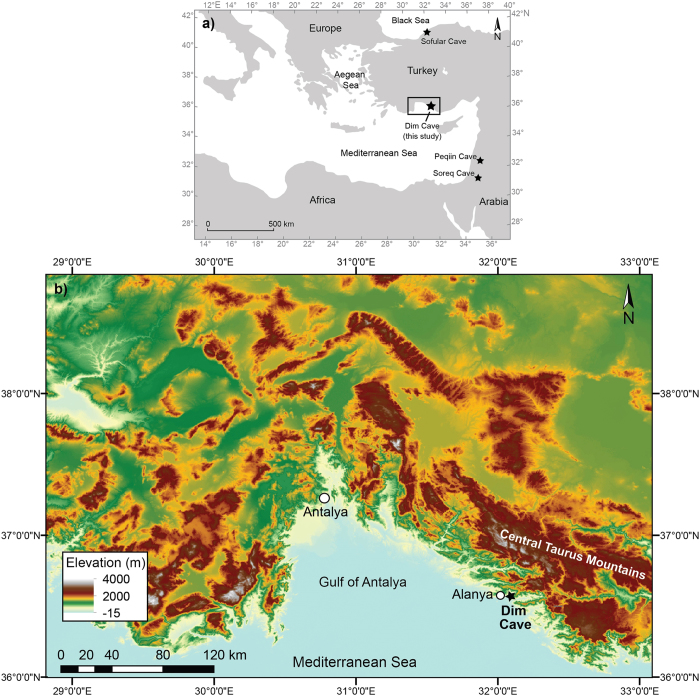
Location map of the Dim Cave, SW Turkey. (**a**) Simplified map of the Eastern Mediterranean region and (**b**) elevation map of SW Turkey, showing the study area. Black stars mark the locations of the Sofular (NW Turkey), Soreq and Peqiin Caves (Israel) in (**a**). Dim Cave is situated at 232 m above sea level. Maps are generated using UQ-licensed software Adobe Illustrator CS5, version 15.0.2 (http://www.adobe.com/au/products/illustrator.html).

The Dim (or locally known as Gavurini) Cave (36°32’N, 32°06’E, 232 m a.s.l.) is located on the western flank of Cebel Reis Mountain (1691 m a.s.l.; Figs 1b and S1a online) of the Central Taurus Mountains and is situated ~6 km from the Mediterranean coast of S-SW Turkey (Fig. 1b). The cave is 360 m long, 10–15 m wide and high and is formed in late Palaeozoic crystalline limestone along a NW-SE trending fault (Fig. S1b^8^ online). The annual mean temperature inside the cave ranges between 18–19 °C with a humidity level of about 90% throughout the year. The modern climate above the cave is typical Mediterranean characterized by dry/hot summers (June-August) and wet/mild winters (December-February), with mean summer (winter) air temperature values of 27 °C; n = 135 (10 °C; n = 140)^9^. Mean precipitation for the Antalya Station (36°52’ N, 30°42’ E, 49 m a.s.l. part of the Global Network of Isotopes in Precipitation (GNIP)) is given as 1075 mm for the period 1963–2009^9^, with 82% (882 mm yr^−1^) and only 1% (12 mm yr^−1^) occurring in the core winter months (when shoulder months are included, this rises to 99%) and summer, respectively. Isotopic data on a monthly basis reveals average winter and summer rainfall δ^18^O values of −6.12‰ and −3.83‰ (VSMOW), respectively (Table S2a online). Most recent data from 2009 shows average δ^18^O value of ~ −7‰ (VSMOW) for rainfall from November to March and no precipitation for the summer months^9^ (Table S2b). Hot/dry summers are due to the northward shift of Intertropical Convergence Zone (ITCZ) into the NH during summer which displaces the sub-tropical anticyclonic (STA) belt to the latitudes of Turkey. In winter, as the ITCZ moves south, westerly air masses derived from the Atlantic penetrate the Mediterranean Basin and bring precipitation to the western and southern coastal margins and mountains of Turkey. Thus, the moisture source for coastal SW Turkey is mostly associated with the intensity and seasonality of NH westerly wind flows and more specifically with Mediterranean Sea storm tracks^10^,^11^. The vegetation cover in the site is characterized by Mediterranean maquis scrublands and pine forests towards the higher altitudes^12^ dominated by C_3_-type vegetation^13^, with very thin soil cover (<20 cm) above the cave.

## Methodology

Three stalagmites from narrow passageways and two water samples from pools of Dim Cave were collected in 2009 and 2012 (Fig. S1 online). Due to mainly financial and logistic constraints, we were unable to establish cave monitoring but cave pool waters were sampled. The stalagmites were halved using a circular saw and micro-sampled along their growth axes using hand drills equipped with diamond-coated drill bits (0.5 to 2 mm in length) for U-series dating (sampling locations reported in Figs S2–S4 online), as well as for stable isotope analyses. Sampling interval for the stable isotope analyses was 0.5–1.0 mm depending on the growth rate of the stalagmite. This sampling interval corresponds to a growth period of 100–200 years for Dim-E3, and an average of ~100 years for Dim-E2 and Dim-E4, respectively. An additional 30 sub-samples (5–6 mg each; 5 samples per layer) were microdrilled from each of the growth phases of Dim-E3 and from one of the growth phases of Dim-E2 and Dim-E4 for Hendy tests^14^. Powder samples (50–100 mg) were analysed for U-series dates in the Radiogenic Isotope Facility (RIF) at the School of Earth Sciences, The University of Queensland (UQ) using a Nu Plasma multi-collector ICP-MS, following the analytical procedure first described in Zhou *et al.* (2011)^15^. Stable isotope analyses for sample powders (3–4 mg) and waters (10 ml) were conducted at the Stable Isotope Geochemistry Laboratory (SIGL) of the School of Earth Sciences (UQ) on an Isoprime Dual Inlet Isotope Ratio Mass Spectrometer (DI-IRMS) with Multiprep. CO_2_ was liberated from carbonate samples by reaction under vacuum with H_3_PO_4_ acid at 90 °C for 1000 s and calibrated against NBS-18 and NBS-19 international standards. The replicate analyses of internal carbonate standards indicate that the analytical standard deviation of δ^18^O and δ^13^C averages at ±0.1‰ (n = 124, 1σ) and ±0.04‰ (n = 124, 1σ) respectively.

## Results

The stalagmite chronologies of the Dim Cave are constructed based on a total of 44 U-series dates (Table S1). 28 ages of stalagmite Dim-E3 (32 cm long) reveal long-term step changes in growth patterns between ~13 and ~90 kyr (Figs. S2 and 2). Similarly, fast-growth stalagmites Dim-E2 (~29 cm long) and Dim-E4 (~38 cm long) show visually recognizable changes in growth patterns in the period from ~10 to ~15 kyr (Figs S3 and S4 online). Age corrections for non-radiogenic ^230^Th contributions are generally very small (Table S1). Micromorphological characteristics of Dim-E2 and -E3 reveal generally similar compact/open columnar calcite fabrics (Fig. S4 and Fig. S5a–e), whereas Dim-E4 is characterized by diagenetically-modified mosaic, dendritic, and open columnar calcite forms^16^ (Figs S4 and S5f online).

**Figure 2:**
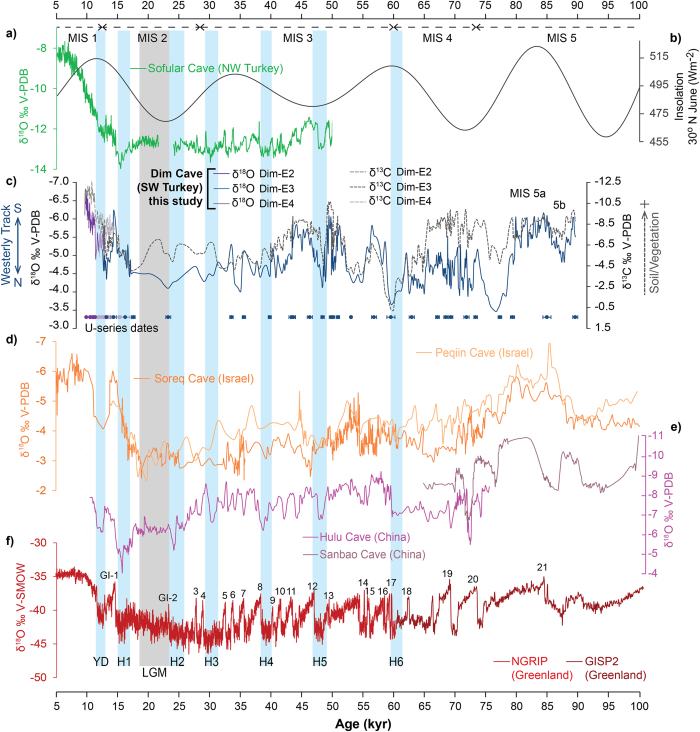
Comparison of the speleothem-based paleoclimatic records over the last 100 kyr. (**a**) δ^18^O time series of stalagmites from Sofular Cave, NW Turkey^5^ (inverted as per that publication); (**b**) summer insolation (30°N June^46^); (**c**) δ^18^O and δ^13^C composite time series of stalagmites Dim-E2, Dim-E3, and Dim-E4 from the Dim Cave (SW Turkey), colour-coded points denote for the U-series dates for Dim-E2 (purple), Dim-E3 (blue) and Dim-E4 (light purple). (**d**) Soreq and Peqiin Cave (Israel) δ^18^O records^4^; (**e**) composite stalagmite δ^18^O records from the Hulu and Sanbao Caves (China)^47^,^48^; (**f**) δ^18^O ice-core time series from Greenland NGRIP^49^ and GISP2^50^, numbers indicating Greenland Interstadials (e.g. GI-1). Marine Isotope Stages (e.g. MIS 1) are indicated by dashed horizontal arrows. Durations of Younger Dryas (YD), Heinrich (H) events, and LGM are highlighted by light blue and grey areas, respectively. Greenland data are provided to constrain Heinrich and other climate events.

A high-resolution stable isotope profile of stalagmite Dim-E3 is based on 442 δ^18^O and δ^13^C measurements (Table S2), which provide a centennial-scale continuous record for the time period ~90–13 kyr. 138 additional high-resolution (Table S2) stable isotope analyses of Dim-E2 and Dim-E4 extend the time series to ~10 kyr for the stalagmite deposition in the Dim Cave. Overall δ^18^O values of the stalagmites range between −3.46 and −6.81‰ VPDB, with the lowest value from Dim-E2 at around 9.9 kyr and highest value from Dim-E3 at around 76.7 kyr (Fig. 2). These are comparable with the results reported from Soreq Cave in Israel for ~100–10 kyr (−6.02 to −2.36‰ VPDB^4^). Overturn rates for the Mediterranean Sea are in the order of 100 years^17^, consequently glacial-interglacial changes in global sea-levels are important. Adjustment of the Dim δ^18^O record for the effect of ice volume-related changes in seawater δ^18^O demonstrate that δ^18^O was reduced by ~1 per mil during the Last Glacial Maximum (LGM) while at Marine Isotope Stage (MIS) 4 the change averages closer to 0.5 per mil between uncorrected and ice volume-corrected records^18^ (Fig. S6). In addition, a temperature-dependent stalagmite calcite and drip water fractionation correction^19^ on Dim-E3 (~90–13 kyr) (Fig. S7) shows that δ^18^O_water_ record has a ~31 per mil (in VSMOW) negative shift, assuming that average temperature was 8 °C degrees lower than today^8^,^20^,^21^ (i.e. MAT 10.5 °C) in the cave, during glacial times. Corrected δ^18^O speleothem values are consistent with the modern precipitation oxygen isotope range (~ −7.5 to −3.5‰^22^,^23^). Corresponding δ^13^C values of Dim speleothems vary broadly from −12.55 (Dim-E2) to −0.21‰ (Dim-E3) VPDB, pointing to a varying degrees of contributions buffered with vegetation/soil-CO_2_ (associated with Mediterranean C_3_-type plants) and dolomitic host limestone (Dim-HR δ^13^C: 5.43 ‰, δ^18^O: −0.71‰ VPDB; Table S3 online) for the seepage waters^4^,^24^,^25^ (Fig. 2; Table S3; Figs. S2–S4). Hendy tests^14^ demonstrate that the δ^18^O values (−6.4 to −4.4‰ VPDB) have not varied by more than 0.5‰ (Fig. S8) and do not correlate (R^2^ = 0.2) with δ^13^C values (−11.3 to −5.08‰ VPDB) along the tested growth laminae (Table S4 online), which means that the deposition of stalagmites was essentially in or close to quasi-isotopic equilibrium, allowing interpretation of the isotopic variations mainly as a climatic indicator in the cave environment.

Modern cave pool waters yield isotopically light δ^18^O and δD ratios (−6.3, −6.2 ±0.1‰ and −29.0 ±2.0‰ VSMOW at 1σ respectively; Fig. S1b), which fall within the range of mean winter rainfall δ^18^O and δD reported from the GNIP Antalya station (δ^18^O: −5.9– −6.2‰ and δD: −22.1– −32.8‰ VSMOW; Table S2a^9^) and are almost identical to the values of δ^18^O and δD of precipitation for the year 2009 (−5.59 and −29.48‰ respectively; Table S2b^9^). When δ^18^O and δD relationships of mean annual and recent 2009 rainfall GNIP data and modern Dim Cave waters are plotted with respect to Mediterranean Meteoric Water Line (MMWL^22^) and Meteoric Water Line (MWL), the majority of isotopic data are observed to fall within the meteoric lines (Fig. S9). While water composition of 2009, mean winter rain (indicated with the numbers 1, 2, 11, and 12; Fig S9) and Dim pool waters lie along the MMWL, summer/spring rain mainly show global isotopic signatures, plotting close to the MWL (Fig. S9).

## Discussion

It is widely recognised that cave environments are complex and a wide range of factors modify isotopic signals of stalagmites. Possible limitations and alternative interpretations have been discussed in the supplementary material (pages: 23–26). Here we summarise the main findings.

Based on the calcite growth rates in Dim-E3 (Fig. S2a), we argue that the moisture balance was net positive above the Dim Cave throughout the last glaciation. Growth rates in Dim-E3 are highest during the interglacial segments and between 72–63 kyr (11.9 mm/kyr). Rates varied between 2.0 mm/kyr at 79–72 kyr through 1.3 mm/kyr at 63–51 kyr to a low of 0.8 mm/kyr between 40 and 18 kyr (Fig. S2a) but deposition continued, which is also inferred from dominant compact/open columnar calcite fabrics^16^ (Fig. S5a–d online). In contrast, after ~8 kyr, speleothem deposition has almost stopped and aragonite has replaced calcite in the speleothems^26^, which is interpreted to reflect a trend toward negative modern water balance^27^.

The heavy winter rain along the Mediterranean coast of Turkey is connected to westerly flows derived from the North Atlantic Ocean but the moisture for the rainfall is mostly sourced from the EM. The close match of the present-day isotopic composition between monitored winter rainfall in Antalya (average δ^18^O and δD values of the months Dec-Feb: −6.12 and −31.3‰ VSMOW respectively; Table S2) and cave pool waters in the Dim Cave (−6.3, −6.2 and −29.0‰ VSMOW; Fig. S1) confirms that the Dim Cave receives mostly winter rain.

**Figure 3:**
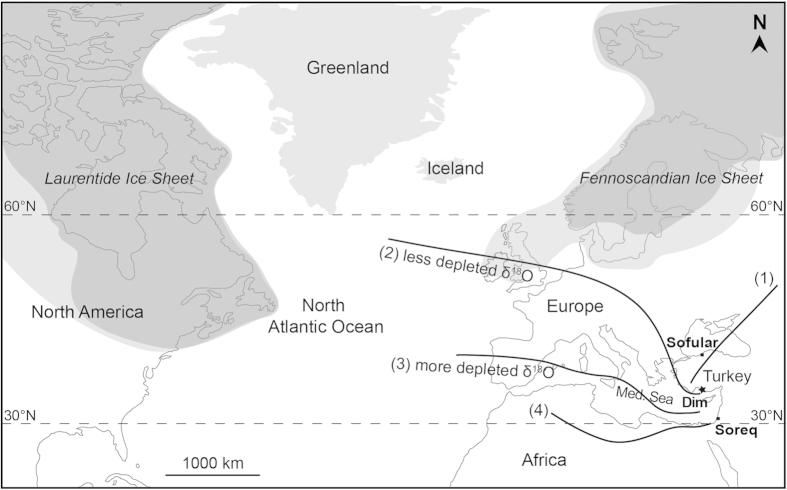
Sketch map of the North Atlantic region showing trajectories of modern precipitating air masses (marked as 1, 2, 3, and 4) over the Eastern Mediterranean^23^,^28^ and changes in ice-sheet volumes during MIS 4 (dark grey) and MIS 2 (light grey shaded areas)^51^,^20^ . The Dim Cave (black star) receives rain through trajectories 2 and 3. Ice limits are indicative only. Map is generated using UQ-licensed software Adobe Illustrator CS5, version 15.0.2 (http://www.adobe.com/au/products/illustrator.html).

Currently the EM region is influenced by four distinct air mass tracks in winter, each characterised by varying moisture content and temperature, depending on the source of the air mass and the extent of interaction with the Mediterranean Sea that modifies the isotopic composition (δ^18^O and δD) of the rain water^23^,^28^. These four air mass trajectories are 1) a NE-SW trajectory coming from Russia, Caucasus and modified in the Turkey region by transit over the Black Sea (d: deuterium excess >22‰); 2) a NW-SE European trajectory across the Balkans (10‰ < d < 22‰); 3) a W-E Mediterranean (or North African coastal) trajectory where the air mass passes over the sea for much of its journey (d < 10‰ and 10‰ < d < 22‰); and 4) an inland African trajectory travelling SW-NE (d < 10‰)^23^,^28^ (Fig. 3). Of these coastal trajectories, Antalya is affected only by 2) and 3) (δ^18^O: −5 to −9‰; δD: ~ −25 to −50‰ SMOW^28^,^29^), as it is protected by major mountain ranges (i.e. Central Taurus Mountains; Fig. 1b) from northerly sourced precipitation and the African trajectory mainly affects areas of the EM far to the south. Based on isotope values of both the rain and pool waters, the Dim Cave is currently primarily affected by W-E precipitation from coastal track 3 (Fig. 3).

Glacial-interglacial δ^18^O changes in the Dim Cave fluctuate from −3.5 to −7‰ VPDB (or equivalently from 27 to 23‰ VSMOW; Table S3) which are typical of other speleothem records from the Mediterranean region (e.g. Soreq and Peqiin Caves^4^). The successful Hendy tests (Fig. S8) allow us to assume that the isotopic fractionation during speleothem carbonate deposition is negligible^30^ despite the fact that some kinetic fractionation via degassing is almost inevitable^31^, and the scale of δ^18^O change in the speleothems is consistent with the scale of δ^18^O changes in air masses (Fig. S7). The variation can be explained as a response to changes in the track of the air mass from a northerly European track across the Balkans that yields less depleted δ^18^O values during MIS 4 to a southerly Mediterranean track that is dominant in interglacials (Fig. 3) and yields depleted values in good agreement with track 3 (Figs. 2 and S2−S4 online). The later part of the last glacial cycle (MIS3/2) appears to demonstrate a progressive but gradual shift from the European track towards the Mediterranean track.

We note that the gradual shift towards isotopically lighter water during the last glaciation would largely disappear if we had not provided an ice volume correction to the δ^18^O results. We argue that this relationship is causal. The intensity of westerly circulation is primarily controlled by pressure (temperature) differences between the North Pole and the tropics. With polar amplification of cooling in glacial times, westerly circulation will intensify during glacial times and especially at times of maximal cooling. In the early part of the glaciation (MIS 4) ice sheet development of both the Laurentide and Fennoscandian sheets is restricted and the polar jet may lie at a northerly position pre-conditioning air mass trajectories largely through the Balkans (Fig. S6). As the glaciation continues, both the Laurentide and Fennoscandian ice-sheets gradually extended southwards (and westward in the case of the Fennoscandian). This increase in surface elevation and pressure forces the polar jet southwards and consequently westerly winds and surface fronts will gradually track further south^32^,^33^ until they adopt a Mediterranean (W-E) like trajectory 3 (Fig. 3). During the Holocene, overall westerly circulation is broadly zonal and for the Dim Cave, the main flows are W-E through the Mediterranean Sea.

The longer-term records appear to reflect multi-millennial scale changes in ice volume, but we also note that those Heinrich events present in the record are also marked by phases of isotopic enrichment. This may reflect either the influx of fresh water into the North Atlantic, changing the isotopic composition of the air mass at the source region and/or enhanced westerly flow during Heinrich events. The enhanced flow would occur because of increased temperature contrast to areas further south and would amplify the effect of the northerly (Balkans) track. This process would be most effective when the continental ice sheets are at their smallest extent (i.e. the YD and MIS 4 events show up most strongly) as there is a northerly track available for the westerlies. During MIS 2, the westerlies may be enhanced but there is little chance for NW-SE flow to dominate because the track of the westerlies is depressed far to the south.

The large range of δ^13^C changes (−12.45 to −0.12‰) in the Dim speleothems (Fig. 2c) can be explained in terms of combined effects of the isotopic signature of the C_3_ vegetation type/soil above the cave and the signature of the dolomitic limestone^30^,^34^. δ^13^C depletion during the interglacial part of the record reflects mixing of soil organic carbon (~ −25‰^35^,^36^) with rain water. In glacial times interaction with soil organic matter was reduced and the δ^13^C was consequently less depleted due to proportionally stronger interactions with the host limestone (δ^13^C: ~5.4‰; Table S3). This reflects thinner density of forest/shrubland cover in the area as a consequence of depressed temperatures and reduced global availability of CO_2_^37^,^38^ during glacial times. Reduced vegetation results in thinner soils and less evapotranspiration at the site. This gives rise to reduced residence time for the waters flowing through epikarst into the cave, and accordingly we observe a combination of more inherited atmospheric CO_2_ (δ^13^C: ~ −7‰^14^,^24^,^39^) and influences from the dolomitic limestone recorded in the speleothems (Fig. 2c and Figs. S2–S4 online). However, it is still important to note that the δ^13^C record shows large positive extensions and strong relationships with δ^18^O during slower growth stages (i.e. marked as slow (s) growth periods of Dim-E3; Fig. S2a). Accordingly we think that degassing (kinetic fractionation) might have played an important role during short and drier periods, but overall we believe that bedrock contribution has an equally important role in the longer term variation of the δ^13^C record.

The Dim isotope record is largely in phase with regional speleothem records from the Soreq and Peqiin Caves^4^ in Israel but is largely out of phase with the Sofular Cave record from NW Turkey^5^. This appears to reflect the strong Mediterranean influence on moisture sources for the Israeli caves and the Dim Cave, whereas the Sofular record is modified by northerly moisture sourced from the Black Sea. Concurrent δ^18^O depletions during interglacial episodes are obvious in both the Israeli and Dim records (Fig. 2c, d). From around 74 to ~41 kyr, the records between Soreq and Dim are apparently out of phase. This is most likely due to relative influences of westerlies at these sites in the early part of the glaciation. The Soreq and Peqiin records display gradual transitions to glacial conditions, whereas the transition is abrupt and significant at the Dim Cave. As the Dim Cave lies many hundreds of kilometres north of the Israeli sites, it is likely to be much more strongly affected by enhancement of the westerly flow as the core of the winter westerly moved south and is also more likely to reflect enhanced NW-SE flow across the Balkans. As the gradual extension of the ice sheets in North America and Europe from MIS4 to MIS2 slowly shifted the westerlies southward, we argue that by the onset of MIS 3, both Dim and the Israeli sites were dominantly controlled by the same air masses (Figs. 2 and 3).

Previous studies have inferred a role of temperature for lighter oxygen isotopes in speleothems during glacial periods and have argued for increased summer rainfall during the late Pleistocene in this region^40^,^41^. This would require the northward migration of the ITCZ beyond Holocene limits at a time when the evidence suggests that major zonal circulations in the northern hemisphere are displaced equatorward. As has already been observed^42^,^43^, this is climatologically not sensible. Part of the reason for the inferences of northward migration of a summer monsoon was that at the Israeli sites, the δ^13^C changes could be explained in part, by inferring more (isotopically less depleted) C_4_ vegetation at glacial times^2^,^27^. At the Dim Cave, there is no C_4_-type vegetation present now^12^ and no reason for C_4_ vegetation (tropical grasses) to occur during glacial times. The isotopically heavier δ^13^C signal is consequently a function of reduced vegetation cover and enhanced bedrock interactions, rather than summer rainfall. Recent research from the Levant region^44^,^45^ has challenged the previous interpretations and proposed that the glacial periods were wetter in response to higher moisture balance, supporting positive moisture balance throughout the last glaciation in the EM. Subsequently, we are the first to deconvolve the glacial pattern in a climatologically sensible manner for SW Turkey and highlight that the records presented here show changes in the westerly trajectories across NW Europe and the Mediterranean.

## Conclusions

In this study, we reconstruct the paleoclimate of the EM region for the past ~80 kyr using a well-dated continuous speleothem record from S-SW Turkey. Our findings demonstrate that the Dim Cave has had a positive moisture balance from ~9–90 kyr. The changes in composite Dim oxygen isotope time series are interpreted as changes in δ^18^O of the oceanic moisture source which is governed by latitudinal shifts in the westerly wind track. Swings in carbon isotope profiles are deduced mostly to reflect changes in the vegetation cover and subsequent interactions with the host dolomitic limestone over glacial-interglacial timescales. Although interrupted by short-term fluctuations, the long-term patterns of the Dim record show synchronous responses to global climatic variations driven by the evolution of the northern hemisphere ice sheets.

## Additional Information

**How to cite this article**: Ünal-İmer, E. *et al.* An 80 kyr-long continuous speleothem record from Dim Cave, SW Turkey with paleoclimatic implications for the Eastern Mediterranean. *Sci. Rep.* **5**, 13560; doi: 10.1038/srep13560 (2015).

## Supplementary Material

Supplementary Information
