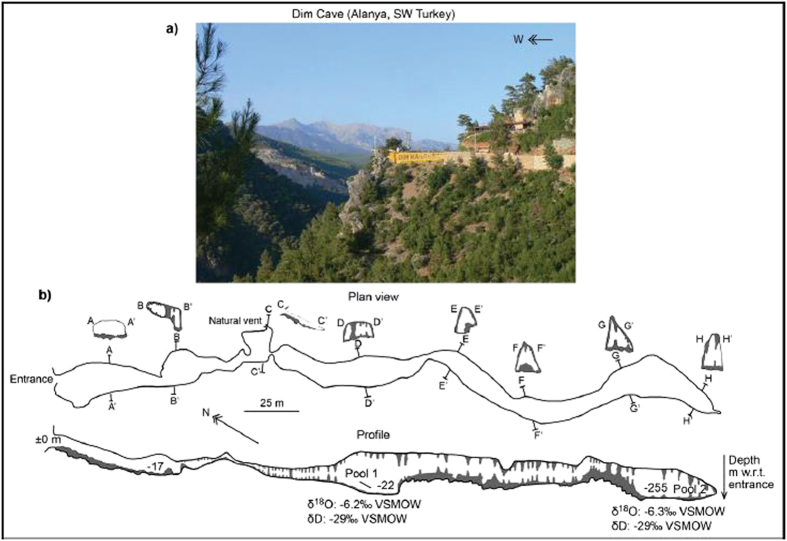# Erratum: An 80 kyr-long continuous speleothem record from Dim Cave, SW Turkey with paleoclimatic implications for the Eastern Mediterranean

**DOI:** 10.1038/srep15595

**Published:** 2015-10-29

**Authors:** Ezgi Ünal-İmer, James Shulmeister, Jian-Xin Zhao, I. Tonguç Uysal, Yue-Xing Feng, Ai Duc Nguyen, Galip Yüce

Scientific Reports 5: Article number: 13560; 10.1038/srep13560published online: 09042015; updated: 10292015

This Article contains a typographical error in Supplementary Figure S1; where ‘Depth mm w.r.t entrance’ should read ‘Depth m w.r.t entrance’. The correct Figure S1 appears below as Fig. 1.

## Figures and Tables

**Figure 1:**