# Supplementary material for: An 80 kyr-long continuous speleothem record from Dim Cave, SW Turkey with paleoclimatic implications for the Eastern Mediterranean
Source: Sci Rep. 2015 Sep 4;5:13560. doi: 10.1038/srep13560 (PMC4559661; doi:10.1038/srep13560)
Supplement: Supplementary Information [file srep13560-s1.pdf]

Supplementary Online Material

An 80 kyr-long continuous speleothem record from Dim Cave, SW Turkey with paleoclimatic implications for the Eastern Mediterranean

by Ezgi Ünal-İmer, James Shulmeister, Jian-Xin Zhao, I. Tonguç Uysal, Yue-Xing Feng, Ai Duc Nguyen, Galip Yüce

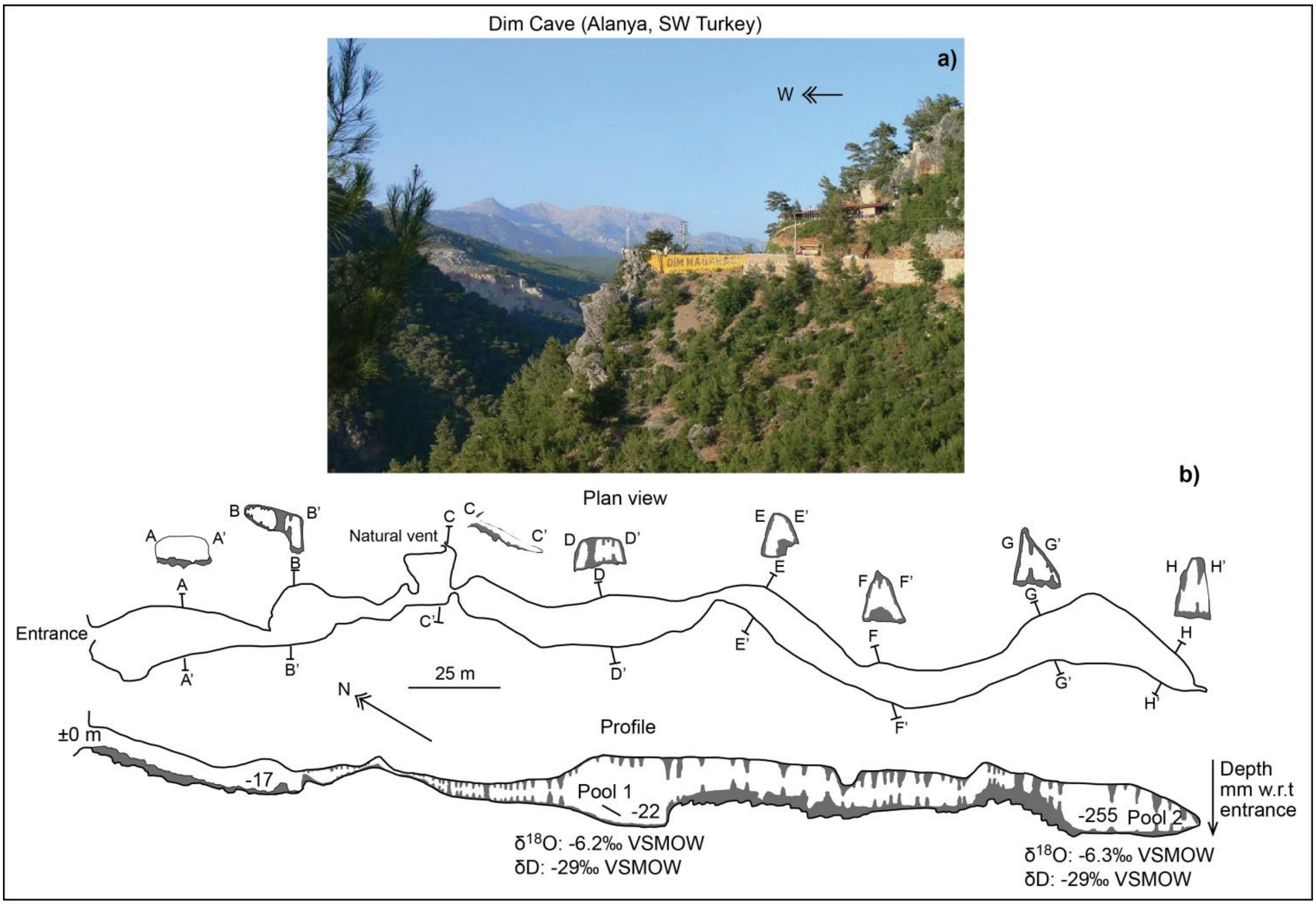

Figure S1 View of Dim Cave looking north **(a)**, plan view and projected profile of Dim Cave with $\delta^{18}O$ and δD data of the pool waters **(b)**. Photograph in (a) is taken by E.U.I. Plan and profile views of the cave in (b) are re-drawn and simplified from an archived report[1] of the General Directorate of Mineral Research and Exploration (Turkey), using the software Adobe Illustrator CS5, version 15.0.2 (http://www.adobe.com/support/downloads/product.jsp?platform=Windows&product=27).

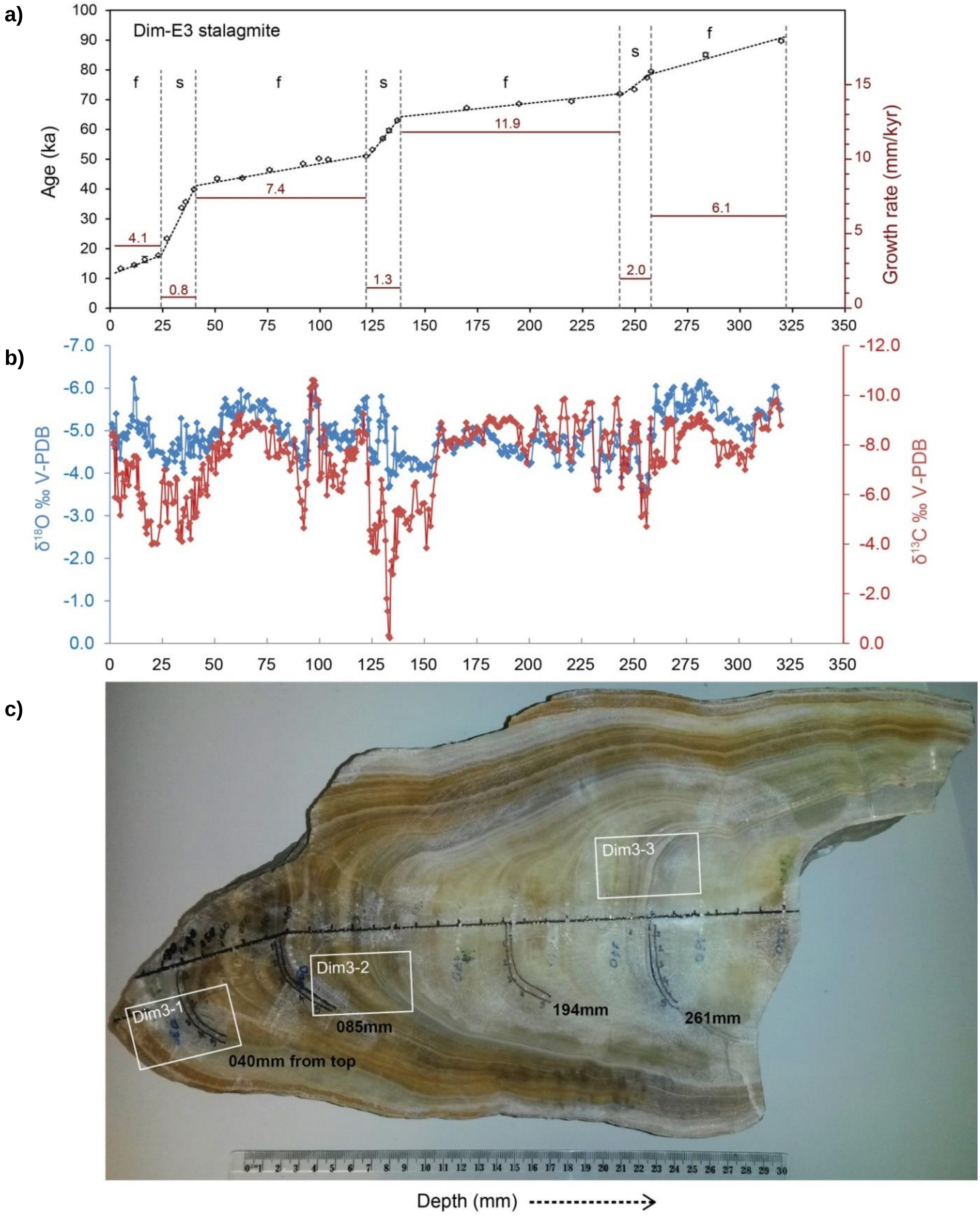

Figure S2 (a) Growth patterns observed in stalagmite Dim-E3 and **(b)** $\delta^{18}O$ and $\delta^{13}C$ isotopic values measured along the axis (mm) of Dim-E3. **(c)** Photo of Dim-E3 showing the Hendy test locations (covering four distinct growth periods: at 040, 085, 194, and 261mm from the tip). White rectangular areas in **(c)** show locations for the thin sections labelled as Dim3-1, -2, and -3. Sudden enrichment in $\delta^{13}C$ (from -9 to -5‰) at ~74 kyr could indicate mixed waters buffered with host dolomitic limestone, forming aragonite layers (Fig. S5c and d) in the speleothem. Possible short hiatuses in Dim-E3 may be associated with lateral offsets and/or sudden color changes at ~77, 60, 48.5, and between 33 and 23 kyr coinciding with the lowest growth rate (0.8mm/kyr; Fig. S2a) during LGM. None of these putative hiatuses are discernible at the resolution of the dating. s: slow, f: fast in **(a)**.

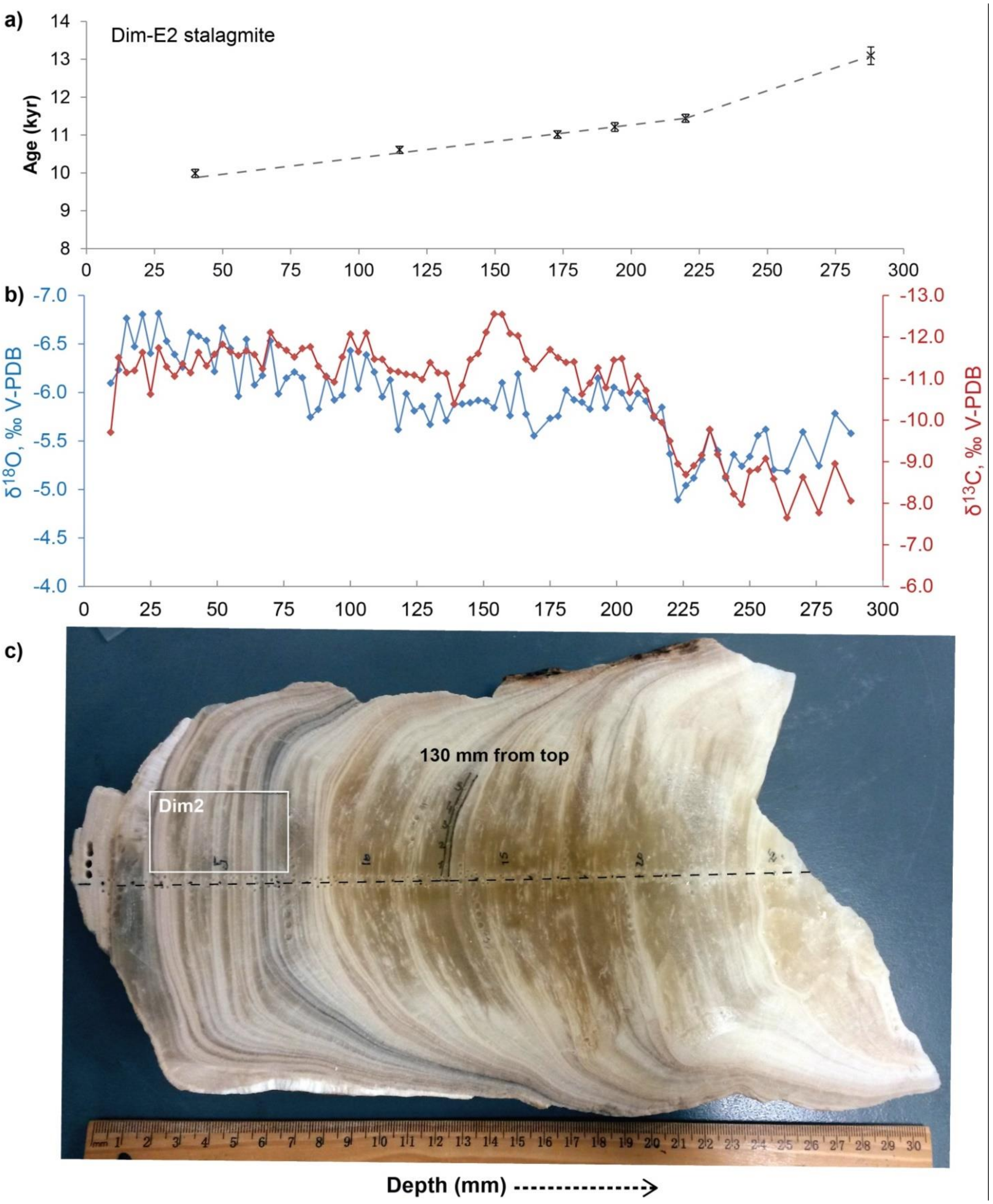

Figure S3 (a) Growth axes of stalagmite Dim-E2 and **(b)** $\delta^{18}O$ and $\delta^{13}C$ isotopic values measured along the axis (mm) of Dim-E2. **(c)** Photo of Dim-E2 showing the Hendy test location at 130mm from the tip. White rectangular area in **(c)** shows location for the thin section: Dim2. Microstratigraphy of Dim-E2 reveals a continuous growth with open/compact columnar calcite with visible banding. Depending on the age-depth model, Dim-E2 suggests fairly continuous growth at relatively constant drip rate except for a possible hiatus at around 11.4 kyr at about 255 mm from the tip.

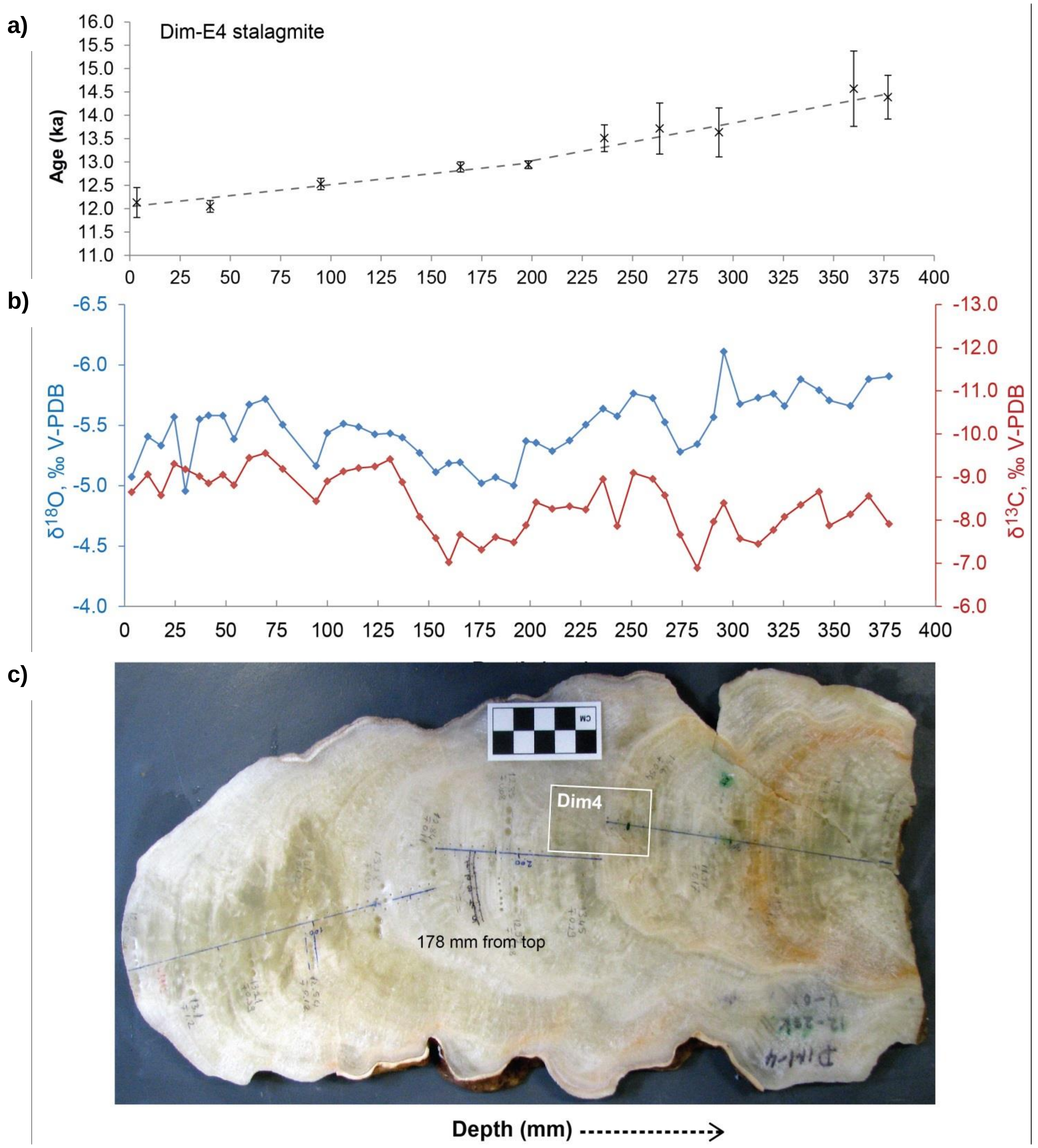

Figure S4 (a) Growth periods observed in stalagmite Dim-E4 and **(b)** $\delta^{18}O$ and $\delta^{13}C$ isotopic values measured along the axis (mm) of Dim-E4. **(c)** Photo of Dim-E4 showing Hendy test location at 178mm from the tip. White rectangular area in **(c)** shows the location for the thin section: Dim4. Microstratigraphy of Dim-E4 shows three distinct growth periods with milky/transparent open columnar and diagenetic mosaic and colloform/dendritic fabrics indicative of seasonal change in drip rate. Rapid-growth Dim-E4 shows two lateral displacements at around 160 and 275 mm from tip corresponding the ages of 12.8 and 13.6 kyr, which may indicate a minor break in growth.

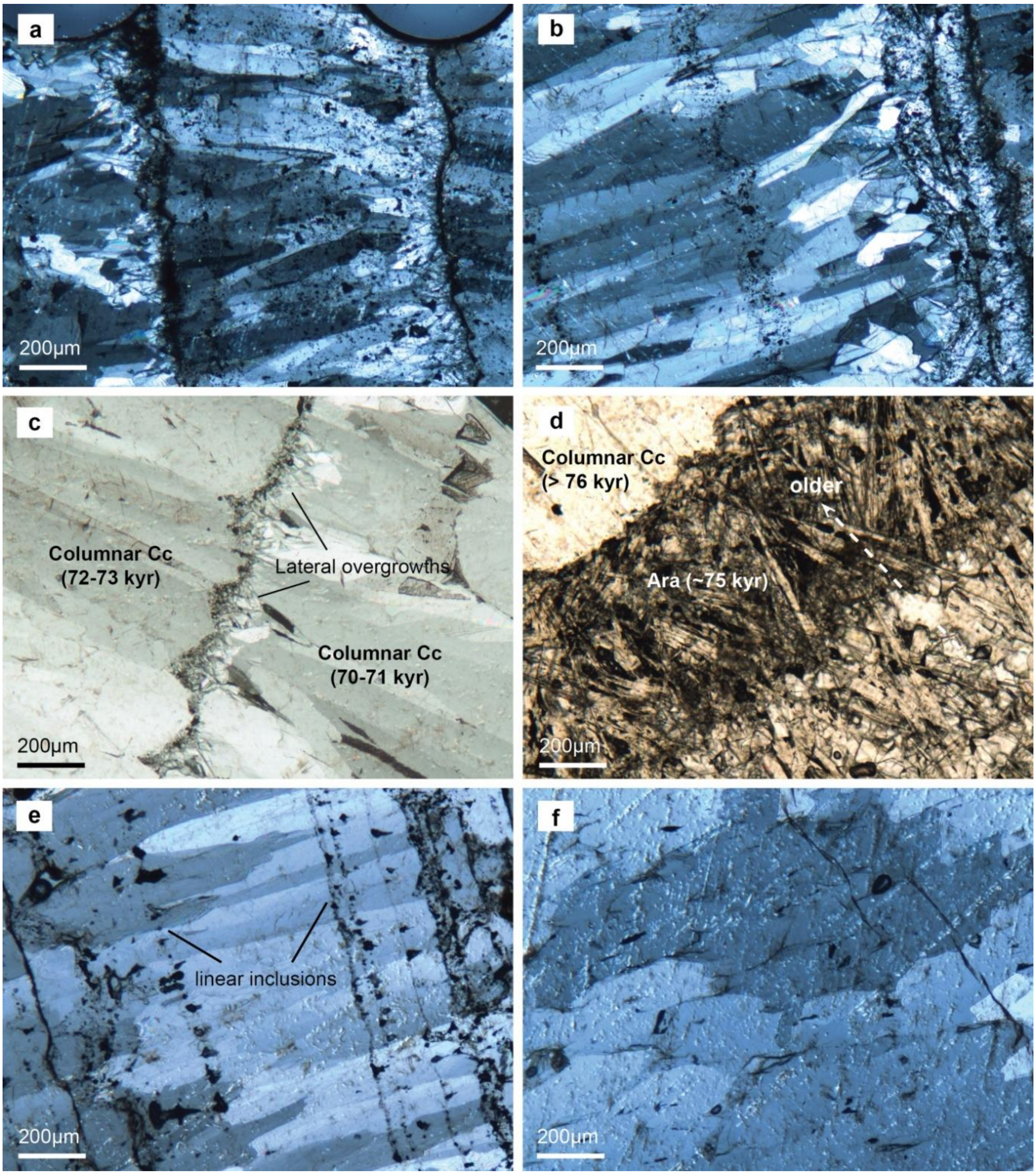

Figure S5 Representative microstructure and calcite fabrics of Dim-E3 (a,b,c, and d), Dim-E2 (e), and Dim-E4 (f) under transmitted light. **a)** Columnar (open) calcite with clear parallel banding and sharp terminations in growth, XPL, from Dim3-1 thin section (marked with rectangular area in Fig. S2c). **b)** Columnar (open) calcite showing intercrystalline boundaries (dark coloured linear inclusions and micritic formations) when growth (or drip rate) is interrupted, XPL, Dim3-2 section in Fig. S2c. **c)** Younger calcite generations and lateral overgrowths in Dim3-3 section, XPL. **d)** Acicular/radiating aragonite (Ara) layers at ~75 kyr with replacement textures towards younger columnar calcite (Cc) layers, from Dim3-3 in Fig.S2c, PPL. **e)** Open/compact columnar calcite indicating relatively constant drip rate, from Dim2 in Fig.S3c, XPL. **f)** Diagenetic mosaic calcite (crystals >30µm)^2 with irregular boundaries (and inclusions), representative of Dim4 thin section (~13 kyr) in Fig. S4c, XPL.

Table S1 MC-ICP-MS Uranium (U)-series age data for stalagmites Dim-E3, Dim-E2, and Dim-E4 from the Dim Cave (Alanya, SW Turkey)

Sample ID	Distance from tip (mm)	U (ppm)	±2σ	^{232}Th (ppb)	±2σ	^{230}Th/^{232}Th	±2σ	^{230}Th/^{238}U	±2σ	^{234}U/^{238}U	±2σ	Uncorr. Age (kyr)	±2σ	*Corr. Age (kyr)	±2σ	Corr. Initial (^{234}U/^{238}U)	±2σ
Dim-E3-0050	5	0.2389	0.0001	6.16	0.007	17.56	0.12	0.1494	0.001	1.2435	0.0016	13.90	0.10	13.28	0.31	1.2546	0.0019
Dim-E3-0115	11.5	0.1756	0.0001	6.56	0.007	12.95	0.07	0.1595	0.0008	1.2106	0.0016	15.35	0.09	14.44	0.45	1.2216	0.002
Dim-E3-0165	16.5	0.1608	0.0001	13.56	0.014	6.9	0.07	0.1919	0.0018	1.2325	0.0023	18.36	0.20	16.3	1.0	1.2491	0.0038
Dim-E3-0225	23	0.075	0.0000	0.98	0.003	63.03	0.78	0.2716	0.0032	1.7733	0.0058	17.92	0.24	17.70	0.26	1.8158	0.0061
Dim-E3-0270	27	0.0767	0.0000	1.98	0.003	34.96	0.45	0.2968	0.0038	1.4943	0.0027	23.85	0.34	23.35	0.41	1.5317	0.0034
Dim-E3-0340	34	0.0997	0.0001	1.69	0.003	68.81	0.42	0.3852	0.0023	1.4198	0.002	34.00	0.24	33.66	0.28	1.4638	0.0024
Dim-E3-0360	36	0.1143	0.0001	0.95	0.002	137.22	0.86	0.3759	0.0023	1.3253	0.0027	35.86	0.27	35.68	0.28	1.3606	0.0029
Dim-E3-0400	40	0.1365	0.0001	2.07	0.002	82.58	0.37	0.4133	0.0018	1.322	0.0018	40.22	0.22	39.89	0.27	1.3619	0.0021
Dim-E3-0510	51	0.1711	0.0001	6.68	0.008	32.14	0.15	0.4137	0.0019	1.2227	0.0015	44.43	0.26	43.50	0.49	1.2545	0.0021
Dim-E3-0630	63	0.2166	0.0001	1.68	0.005	158.54	1.28	0.4043	0.003	1.2092	0.0017	43.88	0.40	43.70	0.41	1.2372	0.0019
Dim-E3-0760	76	0.1901	0.0002	6.88	0.009	37.57	0.13	0.448	0.0014	1.2573	0.0013	47.25	0.20	46.42	0.41	1.2962	0.002
Dim-E3-0920	92	0.1759	0.0002	1.55	0.002	159.61	0.65	0.4629	0.0019	1.2671	0.0016	48.71	0.26	48.51	0.28	1.3071	0.0019
Dim-E3-0995	99.5	0.2043	0.0001	3.55	0.004	77.87	0.32	0.4457	0.0018	1.1869	0.001	50.63	0.26	50.21	0.32	1.2164	0.0013
Dim-E3-1040	104	0.2035	0.0001	0.61	0.001	450.07	2.36	0.4475	0.0022	1.2031	0.0014	49.99	0.32	49.92	0.32	1.234	0.0016
Dim-E3-1220	122	0.2097	0.0002	0.58	0.001	560.68	2.33	0.5103	0.002	1.3405	0.002	51.09	0.27	51.03	0.27	1.3935	0.0022
Dim-E3-1250	125	0.2827	0.0002	0.82	0.001	611.94	1.4	0.5828	0.0011	1.4749	0.0015	53.22	0.14	53.16	0.14	1.5522	0.0016
Dim-E3-1300	130	0.1447	0.0001	0.87	0.002	366.64	2.25	0.7226	0.0042	1.7196	0.0021	56.99	0.43	56.90	0.43	1.8463	0.0025
Dim-E3-1330	133	0.0543	0.0000	1.2	0.002	98.69	0.83	0.7215	0.006	1.6516	0.0021	60.02	0.65	59.64	0.66	1.7757	0.0034
Dim-E3-1370	137	0.1401	0.0001	0.39	0.002	779.07	5.53	0.7125	0.0036	1.5742	0.0018	63.11	0.43	63.06	0.43	1.6867	0.0022
Dim-E3-1700	170	0.1798	0.0001	0.42	0.001	954.21	3.76	0.7277	0.0025	1.5336	0.0011	67.28	0.31	67.24	0.31	1.6457	0.0014
Dim-E3-1950	195	0.1578	0.0001	0.71	0.001	538.62	1.94	0.8008	0.0026	1.6546	0.002	68.69	0.32	68.61	0.32	1.7957	0.0023
Dim-E3-2200	220	0.1744	0.0001	0.13	0.001	3202.78	16.55	0.7673	0.0025	1.5768	0.0018	69.44	0.33	69.43	0.33	1.702	0.0021
Dim-E3-2430	243	0.3062	0.0002	0.77	0.003	926.05	4.85	0.7649	0.003	1.5324	0.0023	72.02	0.42	71.98	0.42	1.6529	0.0027
Dim-E3-2500	250	0.5386	0.0003	0.1	0.001	13369	147	0.7941	0.0023	1.5644	0.001	73.47	0.30	73.47	0.30	1.6944	0.0013
Dim-E3-2560	256	33.6032	0.0245	1.865	0.050	42397	1147	0.7787	0.0021	1.4832	0.0009	77.42	0.30	77.42	0.30	1.6012	0.0011

Dim-E3-2580	258	3.3317	0.0027	0.42	0.005	19598	262	0.8048	0.0024	1.5019	0.001	79.48	0.34	79.48	0.34	1.6281	0.0013
Dim-E3-2840	284	0.1732	0.0001	1.93	0.005	257.96	1.46	0.9471	0.0049	1.6711	0.0025	85.29	0.66	85.11	0.66	1.8562	0.0033
Dim-E3-3200	320	0.284	0.0002	0.84	0.001	925.59	3.09	0.9011	0.0026	1.5432	0.0014	89.77	0.41	89.71	0.41	1.7005	0.0018
Dim-E2-040	40	0.2707	0.0002	0.39	0.01	258.3	6.99	0.1218	0.0013	1.3845	0.0015	10.02	0.11	9.99	0.11	1.3957	0.0015
Dim-E2-115	115	0.2636	0.0001	0.6	0.003	173.69	1.66	0.1309	0.0011	1.4026	0.0018	10.65	0.09	10.61	0.10	1.4151	0.0019
Dim-E2-173	173	0.3733	0.0002	0.27	0.002	557.13	6.28	0.1307	0.0011	1.3555	0.0018	11.03	0.10	11.01	0.10	1.3668	0.0019
Dim-E2-194	194	0.2927	0.0002	0.3	0.002	389.77	4.48	0.1334	0.0013	1.3589	0.0017	11.24	0.12	11.21	0.12	1.3706	0.0018
Dim-E2-220	220	0.2695	0.0002	0.34	0.002	336.98	3.45	0.1409	0.0012	1.4061	0.0021	11.47	0.10	11.44	0.11	1.4196	0.0021
Dim-E2-288	288	0.1262	0.0001	1.21	0.004	51.22	0.79	0.1612	0.0024	1.3992	0.0019	13.30	0.21	13.09	0.23	1.4154	0.0020
Dim-E4-0035	3.5	0.262	0.0002	7.81	0.015	15.36	0.09	0.1509	0.0008	1.3656	0.0011	12.72	0.08	12.08	0.32	1.3813	0.0019
Dim-E4-0400	40	0.2705	0.0001	0.34	0.003	346.19	4.29	0.145	0.0014	1.3768	0.0018	12.09	0.12	12.07	0.12	1.39	0.0019
Dim-E4-0950	95	0.2262	0.0001	1.99	0.004	51.32	0.3	0.1487	0.0008	1.3448	0.0015	12.74	0.08	12.55	0.12	1.3581	0.0016
Dim-E4-1645	164.5	0.2151	0.0001	1.2	0.002	83.63	0.55	0.1544	0.001	1.3728	0.002	12.96	0.09	12.84	0.11	1.3872	0.0020
Dim-E4-1980	198	0.1969	0.0001	0.91	0.001	102.23	0.52	0.1561	0.0008	1.3749	0.0014	13.09	0.07	13.00	0.08	1.3894	0.0014
Dim-E4-2360	236	0.1867	0.0001	4.93	0.005	18.9	0.1	0.1645	0.0009	1.358	0.0015	14.02	0.08	13.45	0.29	1.3746	0.0021
Dim-E4-2635	263.5	0.1886	0.0001	9.55	0.021	10.24	0.07	0.1709	0.0011	1.3439	0.002	14.77	0.10	13.66	0.54	1.3625	0.0033
Dim-E4-2930	293	0.1402	0.0001	6.95	0.018	11	0.14	0.1796	0.0022	1.4193	0.0019	14.68	0.20	13.65	0.53	1.4417	0.0036
Dim-E4-3600	360	0.1322	0.0001	10.19	0.023	7.57	0.08	0.1924	0.0021	1.3841	0.0021	16.24	0.19	14.59	0.81	1.4088	0.0049
Dim-E4-3770	377	0.1545	0.0000	6.8	0.008	12.52	0.11	0.1817	0.0015	1.3851	0.0012	15.26	0.14	14.32	0.47	1.4059	0.0028

Table S2 Monthly precipitation $\delta^{18}O$ and δD means of GNIP Antalya station (36°52'48'' N 30°42' 00'' E; 49 m a.s.l.) from 1963-2009[3] **(a)** and average $\delta^{18}O$, δD, and precipitation (mm) values for 2009 (most recent data from the station) **(b)**

	**Months**	**$\delta^{18}O$‰ (VSMOW)**	**δD‰ (VSMOW)**	**Precipitation (mm)**	**Air Temperature (°C)**	**d-excess value**
	January	-6.23	-32.80	205.4	9.7	18
	February	-5.91	-30.60	159.1	10.3	16.5
	March	-4.89	-25.30	106.9	12.6	14.1
	April	-4.14	-22.40	56.7	16	11.6
	May	-3.17	-18.20	29.5	20.4	7.3
	June	-3.36	-20.60	7.0	25.4	7.5
	July	-4.44	-25.40	3.1	28.4	11.4
	August	-3.70	-18.80	2.2	28.1	10.8
**a)**	September	-2.55	-10.90	12.6	24.6	9.5
	October	-4.66	-23.00	84.1	19.9	15.1
	November	-4.92	-22.10	150.3	14.7	17
	December	-6.21	-30.50	260.2	11.2	19.4
	Lon-term Weighted Annual Means	-5.47	-27.58	-	-	-
	Long-term annual means	-4.52	-23.38	89.76	18.44	13.18

	**Year**	**Avg. $\delta^{18}O$‰ (VSMOW)**	**Avg. δD‰ (VSMOW)**	**Avg. Precipitation (mm)**
**b)**	2009	-5.59	-29.48	1513.7

Table S3 $\delta^{18}O$ and $\delta^{13}C$ stable isotope ratios of the speleothem sub-samples from the Dim Cave (Alanya, SW Turkey) (values are in per mil: ‰)

Sample name	Depth (mm)	$\delta^{13}C$ (VPDB)	$\delta^{18}O$ (VPDB)	$\delta^{18}O$ (VSMOW)
Dim-E3-0010	1	-8.371	-5.157	25.59
Dim-E3-0015	1.5	-8.477	-5.063	25.69
Dim-E3-0020	2	-8.001	-4.726	26.04
Dim-E3-0025	2.5	-5.883	-4.583	26.19
Dim-E3-0030	3	-8.265	-5.398	25.35
Dim-E3-0035	3.5	-6.798	-4.595	26.17
Dim-E3-0040	4	-5.818	-4.845	25.92
Dim-E3-0045	4.5	-5.748	-4.709	26.06
Dim-E3-0050	5	-5.165	-4.342	26.43
Dim-E3-0060	6	-6.845	-4.912	25.85
Dim-E3-0065	6.5	-6.534	-4.928	25.83
Dim-E3-0070	7	-5.912	-4.832	25.93
Dim-E3-0075	7.5	-7.325	-4.861	25.90
Dim-E3-0080	8	-6.611	-4.802	25.96
Dim-E3-0085	8.5	-7.196	-4.951	25.81
Dim-E3-0095	9	-6.366	-5.113	25.64
Dim-E3-0100	10	-7.199	-5.091	25.66
Dim-E3-0105	10.5	-7.254	-5.343	25.40
Dim-E3-0110	11	-7.108	-5.240	25.51
Dim-E3-0115	11.5	-7.533	-6.220	24.50
Dim-E3-0125	12.5	-7.537	-5.756	24.98
Dim-E3-0130	13	-6.283	-5.057	25.70
Dim-E3-0135	13.5	-7.418	-5.192	25.56
Dim-E3-0140	14	-5.977	-4.950	25.81
Dim-E3-0145	14.5	-5.683	-4.996	25.76
Dim-E3-0150	15	-5.435	-5.024	25.73
Dim-E3-0155	15.5	-6.010	-5.143	25.61
Dim-E3-0165	16.5	-5.611	-4.853	25.91
Dim-E3-0170	17	-4.669	-5.134	25.62
Dim-E3-0175	17.5	-4.420	-4.646	26.12
Dim-E3-0180	18	-4.900	-4.710	26.06
Dim-E3-0185	18.5	-4.795	-5.284	25.46
Dim-E3-0195	19.5	-4.915	-4.485	26.29
Dim-E3-0200	20	-3.990	-4.411	26.36
Dim-E3-0205	20.5	-4.034	-4.870	25.89
Dim-E3-0210	21	-4.044	-4.498	26.27
Dim-E3-0225	22.5	-4.016	-4.525	26.25
Dim-E3-0240	24	-4.719	-4.487	26.28
Dim-E3-0250	25	-6.491	-4.481	26.29
Dim-E3-0260	26	-6.988	-4.340	26.44
Dim-E3-0265	26.5	-5.705	-4.188	26.59
Dim-E3-0270	27	-5.638	-4.093	26.69
Dim-E3-0275	27.5	-4.892	-4.249	26.53
Dim-E3-0280	28	-6.432	-4.306	26.47
Dim-E3-0290	29	-6.405	-4.519	26.25
Dim-E3-0300	30	-5.635	-4.690	26.08
Dim-E3-0305	30.5	-5.697	-4.450	26.32
Dim-E3-0310	31	-5.711	-4.277	26.50
Dim-E3-0315	31.5	-6.670	-4.483	26.29
Dim-E3-0320	32	-6.584	-4.446	26.33
Dim-E3-0325	32.5	-4.529	-4.803	25.96
Dim-E3-0330	33	-4.219	-4.654	26.11
Dim-E3-0335	33.5	-4.336	-4.577	26.19
Dim-E3-0340	34	-4.864	-5.291	25.46
Dim-E3-0345	34.5	-4.099	-4.244	26.53
Dim-E3-0350	35	-5.406	-4.119	26.66
Dim-E3-0355	35.5	-5.129	-4.010	26.78

Dim-E3-0365	36.5	-5.869	-5.117	25.64
Dim-E3-0375	37.5	-4.677	-4.453	26.32
Dim-E3-0380	38	-4.839	-4.838	25.92
Dim-E3-0385	38.5	-4.201	-4.399	26.38
Dim-E3-0390	39	-6.109	-4.646	26.12
Dim-E3-0395	39.5	-5.886	-4.714	26.05
Dim-E3-0400	40	-5.077	-4.414	26.36
Dim-E3-0405	40.5	-6.625	-4.865	25.89
Dim-E3-0410	41	-5.094	-4.818	25.94
Dim-E3-0415	41.5	-5.277	-5.183	25.57
Dim-E3-0425	42.5	-6.611	-4.731	26.03
Dim-E3-0430	43	-5.982	-4.380	26.40
Dim-E3-0435	43.5	-6.031	-4.637	26.13
Dim-E3-0440	44	-7.015	-4.996	25.76
Dim-E3-0445	44.5	-7.215	-4.799	25.96
Dim-E3-0455	45.5	-6.191	-4.583	26.19
Dim-E3-0460	46	-6.369	-4.502	26.27
Dim-E3-0465	46.5	-5.957	-4.564	26.21
Dim-E3-0470	47	-7.493	-4.766	26.00
Dim-E3-0475	47.5	-7.376	-4.584	26.18
Dim-E3-0480	48	-7.318	-4.880	25.88
Dim-E3-0485	48.5	-7.021	-4.738	26.03
Dim-E3-0490	49	-7.938	-4.960	25.80
Dim-E3-0500	50	-7.886	-5.036	25.72
Dim-E3-0505	50.5	-6.846	-4.889	25.87
Dim-E3-0510	51	-7.832	-4.994	25.76
Dim-E3-0515	51.5	-8.072	-4.946	25.81
Dim-E3-0520	52	-7.424	-4.886	25.87
Dim-E3-0525	52.5	-8.058	-4.996	25.76
Dim-E3-0530	53	-8.486	-5.371	25.37
Dim-E3-0535	53.5	-8.057	-4.938	25.82
Dim-E3-0540	54	-7.541	-5.530	25.21
Dim-E3-0545	54.5	-7.748	-5.564	25.17
Dim-E3-0550	55	-7.673	-5.746	24.99
Dim-E3-0555	55.5	-7.715	-5.613	25.12
Dim-E3-0560	56	-7.647	-5.389	25.35
Dim-E3-0565	56.5	-8.015	-5.542	25.20
Dim-E3-0570	57	-7.573	-5.461	25.28
Dim-E3-0575	57.5	-8.034	-5.360	25.39
Dim-E3-0580	58	-8.403	-5.366	25.38
Dim-E3-0585	58.5	-8.419	-5.332	25.41
Dim-E3-0590	59	-8.838	-5.552	25.19
Dim-E3-0595	59.5	-8.482	-5.334	25.41
Dim-E3-0600	60	-8.780	-5.219	25.53
Dim-E3-0610	61	-9.125	-5.655	25.08
Dim-E3-0615	61.5	-8.876	-5.423	25.32
Dim-E3-0620	62	-8.797	-5.318	25.43
Dim-E3-0625	62.5	-9.221	-5.951	24.78
Dim-E3-0630	63	-8.347	-5.446	25.30
Dim-E3-0640	64	-8.489	-5.535	25.20
Dim-E3-0650	65	-8.809	-5.790	24.94
Dim-E3-0660	66	-8.612	-5.830	24.90
Dim-E3-0670	67	-8.634	-5.481	25.26
Dim-E3-0680	68	-8.901	-5.562	25.18
Dim-E3-0690	69	-8.845	-5.548	25.19
Dim-E3-0700	70	-8.814	-5.108	25.64
Dim-E3-0710	71	-8.945	-5.695	25.04
Dim-E3-0720	72	-8.612	-5.476	25.27
Dim-E3-0730	73	-8.690	-5.620	25.12
Dim-E3-0740	74	-8.666	-5.714	25.02
Dim-E3-0750	75	-7.716	-5.536	25.20

Dim-E3-0760	76	-7.502	-5.272	25.48
Dim-E3-0770	77	-8.529	-5.456	25.29
Dim-E3-0780	78	-8.728	-5.465	25.28
Dim-E3-0790	79	-7.528	-5.225	25.52
Dim-E3-0800	80	-7.617	-5.085	25.67
Dim-E3-0805	80.5	-7.489	-4.945	25.81
Dim-E3-0810	81	-8.186	-5.196	25.55
Dim-E3-0820	82	-7.767	-4.822	25.94
Dim-E3-0830	83	-7.716	-4.903	25.86
Dim-E3-0840	84	-7.988	-4.967	25.79
Dim-E3-0850	85	-7.668	-5.130	25.62
Dim-E3-0860	86	-6.968	-4.704	26.06
Dim-E3-0870	87	-7.281	-4.677	26.09
Dim-E3-0880	88	-8.229	-4.745	26.02
Dim-E3-0890	89	-7.480	-4.580	26.19
Dim-E3-0900	90	-6.258	-4.406	26.37
Dim-E3-0910	91	-5.697	-4.287	26.49
Dim-E3-0915	91.5	-5.745	-4.112	26.67
Dim-E3-0920	92	-5.057	-4.155	26.63
Dim-E3-0925	92.5	-4.650	-4.713	26.05
Dim-E3-0930	93	-5.385	-4.431	26.34
Dim-E3-0935	93.5	-6.501	-5.211	25.54
Dim-E3-0940	94	-6.404	-4.308	26.47
Dim-E3-0945	94.5	-7.186	-4.528	26.24
Dim-E3-0950	95	-8.680	-4.973	25.78
Dim-E3-0955	95.5	-10.28	-5.818	24.91
Dim-E3-0960	96	-9.427	-5.037	25.72
Dim-E3-0965	96.5	-10.61	-6.057	24.67
Dim-E3-0970	97	-10.34	-5.767	24.97
Dim-E3-0975	97.5	-10.59	-5.723	25.01
Dim-E3-0980	98	-10.42	-6.047	24.68
Dim-E3-0985	98.5	-9.857	-5.578	25.16
Dim-E3-0995	99.5	-9.789	-5.622	25.11
Dim-E3-1000	100	-6.586	-4.804	25.96
Dim-E3-1005	100.5	-6.675	-4.627	26.14
Dim-E3-1010	101	-6.855	-4.679	26.09
Dim-E3-1015	101.5	-7.864	-4.855	25.91
Dim-E3-1020	102	-8.613	-5.136	25.62
Dim-E3-1025	102.5	-8.264	-5.080	25.67
Dim-E3-1030	103	-7.970	-5.059	25.70
Dim-E3-1035	103.5	-5.969	-4.461	26.31
Dim-E3-1040	104	-6.717	-4.963	25.79
Dim-E3-1050	105	-7.606	-5.110	25.64
Dim-E3-1055	105.5	-6.925	-4.908	25.85
Dim-E3-1060	106	-6.724	-4.832	25.93
Dim-E3-1065	106.5	-6.333	-4.863	25.90
Dim-E3-1070	107	-7.166	-4.958	25.80
Dim-E3-1075	107.5	-6.816	-5.159	25.59
Dim-E3-1080	108	-6.775	-4.916	25.84
Dim-E3-1085	108.5	-6.752	-4.830	25.93
Dim-E3-1095	109	-6.182	-4.607	26.16
Dim-E3-1100	110	-6.185	-4.561	26.21
Dim-E3-1105	110.5	-6.116	-4.774	25.99
Dim-E3-1110	111	-6.450	-4.828	25.93
Dim-E3-1115	111.5	-6.968	-4.917	25.84
Dim-E3-1120	112	-6.911	-4.932	25.83
Dim-E3-1125	112.5	-7.332	-4.824	25.94
Dim-E3-1130	113	-7.763	-4.969	25.79
Dim-E3-1135	113.5	-7.636	-4.768	25.99
Dim-E3-1140	114	-7.535	-4.598	26.17
Dim-E3-1145	114.5	-7.624	-4.627	26.14

Dim-E3-1150	115	-7.428	-4.767	26.00
Dim-E3-1155	115.5	-7.822	-4.964	25.79
Dim-E3-1160	116	-7.563	-4.806	25.96
Dim-E3-1165	116.5	-7.429	-4.910	25.85
Dim-E3-1170	117	-7.848	-5.251	25.50
Dim-E3-1175	117.5	-7.721	-5.230	25.52
Dim-E3-1180	118	-8.510	-5.484	25.26
Dim-E3-1185	118.5	-8.510	-5.539	25.20
Dim-E3-1190	119	-8.549	-5.453	25.29
Dim-E3-1200	120	-8.095	-5.322	25.42
Dim-E3-1205	120.5	-8.183	-5.131	25.62
Dim-E3-1210	121	-9.236	-5.720	25.01
Dim-E3-1215	121.5	-8.470	-5.552	25.19
Dim-E3-1220	122	-8.428	-5.543	25.20
Dim-E3-1225	122.5	-8.391	-5.780	24.95
Dim-E3-1230	123	-6.470	-4.996	25.76
Dim-E3-1235	123.5	-7.238	-5.259	25.49
Dim-E3-1240	124	-4.557	-4.576	26.19
Dim-E3-1245	124.5	-4.085	-4.538	26.23
Dim-E3-1250	125	-4.319	-4.358	26.42
Dim-E3-1255	125.5	-3.706	-4.241	26.54
Dim-E3-1260	126	-4.548	-4.334	26.44
Dim-E3-1265	126.5	-4.767	-4.466	26.31
Dim-E3-1270	127	-3.666	-4.272	26.51
Dim-E3-1275	127.5	-3.723	-4.266	26.51
Dim-E3-1280	128	-4.673	-4.755	26.01
Dim-E3-1285	128.5	-5.272	-5.224	25.53
Dim-E3-1290	129	-4.763	-5.088	25.67
Dim-E3-1295	129.5	-6.584	-5.804	24.93
Dim-E3-1300	130	-6.207	-5.536	25.20
Dim-E3-1305	130.5	-5.655	-5.473	25.27
Dim-E3-1310	131	-4.917	-5.211	25.54
Dim-E3-1315	131.5	-4.143	-4.753	26.01
Dim-E3-1320	132	-1.798	-5.360	25.38
Dim-E3-1325	132.5	-1.295	-4.090	26.69
Dim-E3-1330	133	-0.311	-3.658	27.14
Dim-E3-1335	133.5	-0.218	-3.708	27.09
Dim-E3-1340	134	-2.928	-3.967	26.82
Dim-E3-1345	134.5	-3.313	-4.021	26.76
Dim-E3-1350	135	-2.783	-4.030	26.76
Dim-E3-1355	135.5	-3.781	-4.443	26.33
Dim-E3-1360	136	-5.329	-5.054	25.70
Dim-E3-1365	136.5	-3.465	-3.960	26.83
Dim-E3-1370	137	-4.074	-4.417	26.36
Dim-E3-1375	137.5	-5.168	-4.327	26.45
Dim-E3-1380	138	-5.432	-4.392	26.38
Dim-E3-1390	139	-5.166	-4.177	26.60
Dim-E3-1400	140	-5.302	-4.472	26.30
Dim-E3-1410	141	-4.829	-4.303	26.47
Dim-E3-1420	142	-4.967	-4.356	26.42
Dim-E3-1430	143	-4.582	-4.340	26.44
Dim-E3-1440	144	-5.114	-4.089	26.69
Dim-E3-1450	145	-6.346	-4.342	26.43
Dim-E3-1460	146	-6.482	-4.319	26.46
Dim-E3-1470	147	-5.323	-4.097	26.69
Dim-E3-1480	148	-5.279	-4.213	26.57
Dim-E3-1490	149	-5.619	-4.135	26.65
Dim-E3-1500	150	-5.643	-4.086	26.70
Dim-E3-1510	151	-3.847	-4.199	26.58
Dim-E3-1520	152	-5.394	-4.131	26.65
Dim-E3-1530	153	-4.719	-3.939	26.85

Dim-E3-1540	154	-5.963	-4.283	26.50
Dim-E3-1550	155	-6.781	-4.665	26.10
Dim-E3-1560	156	-7.505	-4.708	26.06
Dim-E3-1570	157	-8.402	-5.053	25.70
Dim-E3-1580	158	-8.891	-5.160	25.59
Dim-E3-1590	159	-8.598	-4.777	25.99
Dim-E3-1600	160	-7.774	-4.529	26.24
Dim-E3-1610	161	-8.084	-4.731	26.03
Dim-E3-1620	162	-8.147	-4.696	26.07
Dim-E3-1630	163	-7.888	-4.680	26.09
Dim-E3-1640	164	-8.258	-4.470	26.30
Dim-E3-1650	165	-8.235	-4.597	26.17
Dim-E3-1660	166	-8.096	-4.673	26.09
Dim-E3-1670	167	-8.123	-4.742	26.02
Dim-E3-1680	168	-8.070	-4.711	26.05
Dim-E3-1690	169	-8.291	-5.002	25.75
Dim-E3-1700	170	-8.895	-4.649	26.12
Dim-E3-1710	171	-8.661	-4.758	26.01
Dim-E3-1720	172	-8.198	-4.705	26.06
Dim-E3-1730	173	-8.417	-5.069	25.69
Dim-E3-1740	174	-8.434	-4.832	25.93
Dim-E3-1750	175	-8.709	-4.977	25.78
Dim-E3-1760	176	-8.584	-4.768	26.00
Dim-E3-1770	177	-8.546	-4.960	25.80
Dim-E3-1780	178	-8.046	-4.760	26.00
Dim-E3-1790	179	-8.808	-5.070	25.68
Dim-E3-1800	180	-8.916	-5.000	25.76
Dim-E3-1810	181	-9.124	-4.923	25.84
Dim-E3-1820	182	-8.962	-4.828	25.93
Dim-E3-1830	183	-9.039	-4.890	25.87
Dim-E3-1840	184	-8.837	-4.769	25.99
Dim-E3-1850	185	-8.714	-4.734	26.03
Dim-E3-1860	186	-8.735	-4.514	26.26
Dim-E3-1870	187	-8.913	-4.440	26.33
Dim-E3-1880	188	-9.048	-4.626	26.14
Dim-E3-1890	189	-8.954	-4.514	26.26
Dim-E3-1900	190	-9.011	-4.401	26.37
Dim-E3-1910	191	-9.104	-4.633	26.13
Dim-E3-1920	192	-8.975	-4.512	26.26
Dim-E3-1930	193	-8.934	-4.514	26.26
Dim-E3-1940	194	-8.846	-4.641	26.13
Dim-E3-1950	195	-8.071	-4.367	26.41
Dim-E3-1960	196	-8.698	-4.346	26.43
Dim-E3-1970	197	-7.817	-4.457	26.32
Dim-E3-1980	198	-7.758	-4.429	26.34
Dim-E3-1990	199	-7.250	-4.270	26.51
Dim-E3-2000	200	-7.986	-4.197	26.58
Dim-E3-2010	201	-8.348	-4.250	26.53
Dim-E3-2020	202	-8.389	-4.755	26.01
Dim-E3-2030	203	-8.602	-4.794	25.97
Dim-E3-2040	204	-9.497	-5.522	25.22
Dim-E3-2050	205	-9.357	-4.750	26.01
Dim-E3-2060	206	-9.013	-4.910	25.85
Dim-E3-2070	207	-9.100	-4.894	25.86
Dim-E3-2080	208	-8.749	-4.650	26.12
Dim-E3-2090	209	-8.405	-4.743	26.02
Dim-E3-2100	210	-7.962	-4.670	26.10
Dim-E3-2110	211	-8.250	-4.613	26.15
Dim-E3-2120	212	-8.426	-4.483	26.29
Dim-E3-2130	213	-7.780	-4.172	26.61
Dim-E3-2140	214	-7.788	-4.184	26.60

Dim-E3-2150	215	-9.086	-4.723	26.04
Dim-E3-2160	216	-9.800	-4.865	25.90
Dim-E3-2170	217	-9.853	-4.854	25.91
Dim-E3-2180	218	-9.077	-4.679	26.09
Dim-E3-2190	219	-7.581	-4.244	26.54
Dim-E3-2200	220	-7.346	-4.080	26.70
Dim-E3-2210	221	-7.836	-4.246	26.53
Dim-E3-2220	222	-9.145	-4.842	25.92
Dim-E3-2230	223	-8.975	-4.654	26.11
Dim-E3-2240	224	-8.540	-4.478	26.29
Dim-E3-2250	225	-8.255	-4.435	26.34
Dim-E3-2260	226	-8.999	-4.926	25.83
Dim-E3-2270	227	-9.308	-4.961	25.80
Dim-E3-2280	228	-9.672	-4.986	25.77
Dim-E3-2290	229	-9.673	-5.114	25.64
Dim-E3-2300	230	-9.685	-5.203	25.55
Dim-E3-2310	231	-6.978	-4.119	26.66
Dim-E3-2320	232	-6.191	-4.068	26.72
Dim-E3-2330	233	-6.205	-3.908	26.88
Dim-E3-2340	234	-7.764	-5.282	25.47
Dim-E3-2350	235	-8.267	-4.429	26.34
Dim-E3-2360	236	-8.535	-4.308	26.47
Dim-E3-2370	237	-8.436	-4.386	26.39
Dim-E3-2380	238	-7.520	-4.048	26.74
Dim-E3-2390	239	-8.094	-4.340	26.44
Dim-E3-2400	240	-8.497	-4.427	26.35
Dim-E3-2410	241	-9.647	-4.909	25.85
Dim-E3-2420	242	-9.871	-4.650	26.12
Dim-E3-2430	243	-9.003	-5.129	25.62
Dim-E3-2440	244	-6.281	-3.901	26.89
Dim-E3-2445	244.5	-7.001	-4.194	26.59
Dim-E3-2450	245	-7.884	-4.363	26.41
Dim-E3-2455	245.5	-7.022	-4.350	26.43
Dim-E3-2460	246	-7.052	-4.116	26.67
Dim-E3-2465	246.5	-7.406	-4.207	26.57
Dim-E3-2470	247	-6.936	-4.032	26.75
Dim-E3-2475	247.5	-7.757	-4.069	26.72
Dim-E3-2480	248	-7.823	-4.114	26.67
Dim-E3-2485	248.5	-8.156	-4.701	26.06
Dim-E3-2490	249	-8.304	-4.889	25.87
Dim-E3-2495	249.5	-8.718	-4.809	25.95
Dim-E3-2500	250	-8.911	-5.098	25.65
Dim-E3-2505	250.5	-8.622	-5.156	25.60
Dim-E3-2510	251	-8.295	-4.716	26.05
Dim-E3-2515	251.5	-8.216	-4.360	26.42
Dim-E3-2520	252	-8.835	-4.263	26.52
Dim-E3-2525	252.5	-8.122	-3.934	26.85
Dim-E3-2530	253	-6.733	-3.874	26.92
Dim-E3-2535	253.5	-5.096	-3.652	27.15
Dim-E3-2540	254	-6.382	-3.552	27.25
Dim-E3-2550	255	-5.942	-3.455	27.35
Dim-E3-2555	255.5	-5.238	-3.531	27.27
Dim-E3-2560	256	-4.706	-3.653	27.14
Dim-E3-2565	256.5	-6.061	-3.954	26.83
Dim-E3-2570	257	-6.189	-3.885	26.91
Dim-E3-2575	257.5	-8.241	-4.869	25.89
Dim-E3-2580	258	-9.066	-5.038	25.72
Dim-E3-2585	258.5	-7.119	-4.755	26.01
Dim-E3-2590	259	-7.392	-4.842	25.92
Dim-E3-2595	259.5	-7.235	-4.898	25.86
Dim-E3-2600	260	-6.864	-5.535	25.20

Dim-E3-2605	260.5	-7.701	-6.048	24.68
Dim-E3-2610	261	-7.735	-5.491	25.25
Dim-E3-2615	261.5	-7.414	-5.672	25.06
Dim-E3-2620	262	-7.123	-5.504	25.24
Dim-E3-2625	262.5	-7.358	-5.522	25.22
Dim-E3-2630	263	-7.444	-5.428	25.31
Dim-E3-2635	263.5	-7.324	-5.388	25.36
Dim-E3-2640	264	-7.183	-5.209	25.54
Dim-E3-2645	264.5	-7.283	-5.200	25.55
Dim-E3-2650	265	-8.658	-5.620	25.12
Dim-E3-2655	265.5	-9.038	-5.670	25.07
Dim-E3-2660	266	-8.758	-5.832	24.90
Dim-E3-2665	266.5	-8.818	-5.774	24.96
Dim-E3-2670	267	-8.952	-5.785	24.95
Dim-E3-2675	267.5	-9.066	-5.846	24.88
Dim-E3-2680	268	-8.842	-5.961	24.77
Dim-E3-2690	269	-8.937	-6.022	24.70
Dim-E3-2695	269.5	-7.367	-5.221	25.53
Dim-E3-2700	270	-7.332	-5.266	25.48
Dim-E3-2705	270.5	-7.896	-5.632	25.10
Dim-E3-2710	271	-7.875	-5.584	25.15
Dim-E3-2715	271.5	-8.263	-5.617	25.12
Dim-E3-2720	272	-8.465	-5.443	25.30
Dim-E3-2725	272.5	-8.425	-5.566	25.17
Dim-E3-2730	273	-8.416	-5.453	25.29
Dim-E3-2735	273.5	-8.551	-5.587	25.15
Dim-E3-2740	274	-8.674	-5.987	24.74
Dim-E3-2745	274.5	-8.834	-6.060	24.66
Dim-E3-2750	275	-8.719	-5.881	24.85
Dim-E3-2755	275.5	-8.776	-5.608	25.13
Dim-E3-2760	276	-8.758	-5.485	25.26
Dim-E3-2765	276.5	-8.777	-5.500	25.24
Dim-E3-2770	277	-8.723	-5.716	25.02
Dim-E3-2775	277.5	-9.091	-5.928	24.80
Dim-E3-2780	278	-8.813	-5.617	25.12
Dim-E3-2785	278.5	-8.806	-5.820	24.91
Dim-E3-2790	279	-8.821	-5.734	25.00
Dim-E3-2800	280	-8.984	-5.997	24.73
Dim-E3-2805	280.5	-9.116	-5.996	24.73
Dim-E3-2810	281	-9.214	-6.097	24.63
Dim-E3-2815	281.5	-8.893	-6.159	24.56
Dim-E3-2820	282	-9.230	-6.085	24.64
Dim-E3-2830	283	-8.669	-5.641	25.10
Dim-E3-2840	284	-8.954	-6.083	24.64
Dim-E3-2850	285	-8.724	-5.796	24.94
Dim-E3-2860	286	-8.679	-5.894	24.83
Dim-E3-2870	287	-8.609	-5.468	25.27
Dim-E3-2880	288	-8.132	-5.757	24.98
Dim-E3-2890	289	-8.123	-5.704	25.03
Dim-E3-2900	290	-7.179	-5.280	25.47
Dim-E3-2910	291	-7.449	-5.296	25.45
Dim-E3-2920	292	-8.055	-5.338	25.41
Dim-E3-2930	293	-8.096	-5.385	25.36
Dim-E3-2940	294	-7.584	-5.231	25.52
Dim-E3-2950	295	-7.531	-5.187	25.56
Dim-E3-2960	296	-8.092	-5.501	25.24
Dim-E3-2970	297	-7.650	-5.207	25.54
Dim-E3-2980	298	-7.887	-5.263	25.48
Dim-E3-2990	299	-7.927	-5.271	25.48
Dim-E3-3000	300	-7.237	-5.045	25.71
Dim-E3-3010	301	-7.618	-5.119	25.63

Dim-E3-3020	302	-7.477	-4.925	25.83
Dim-E3-3030	303	-6.993	-4.809	25.95
Dim-E3-3040	304	-7.777	-5.089	25.66
Dim-E3-3050	305	-7.765	-5.048	25.71
Dim-E3-3060	306	-7.528	-4.805	25.96
Dim-E3-3070	307	-7.942	-5.147	25.60
Dim-E3-3080	308	-9.042	-5.348	25.40
Dim-E3-3090	309	-9.198	-5.494	25.25
Dim-E3-3100	310	-9.221	-5.349	25.40
Dim-E3-3110	311	-8.646	-5.439	25.30
Dim-E3-3120	312	-8.667	-5.371	25.37
Dim-E3-3130	313	-8.722	-5.284	25.46
Dim-E3-3140	314	-8.611	-5.286	25.46
Dim-E3-3150	315	-9.299	-5.717	25.02
Dim-E3-3160	316	-9.633	-5.695	25.04
Dim-E3-3170	317	-9.702	-6.036	24.69
Dim-E3-3180	318	-9.812	-6.017	24.71
Dim-E3-3190	319	-9.480	-5.635	25.10
Dim-E3-3200	320	-8.779	-5.499	25.24
Dim-E2-010	10	-9.709	-6.095	24.63
Dim-E2-013	13	-11.51	-6.233	24.48
Dim-E2-016	16	-11.14	-6.764	23.94
Dim-E2-019	19	-11.19	-6.472	24.24
Dim-E2-022	22	-11.63	-6.804	23.90
Dim-E2-025	25	-10.63	-6.403	24.31
Dim-E2-028	28	-11.74	-6.814	23.89
Dim-E2-031	31	-11.29	-6.528	24.18
Dim-E2-034	34	-11.05	-6.391	24.32
Dim-E2-037	37	-11.35	-6.260	24.46
Dim-E2-040	40	-11.14	-6.617	24.09
Dim-E2-043	43	-11.63	-6.580	24.13
Dim-E2-046	46	-11.31	-6.536	24.17
Dim-E2-049	49	-11.58	-6.216	24.50
Dim-E2-052	52	-11.83	-6.665	24.04
Dim-E2-055	55	-11.65	-6.454	24.26
Dim-E2-058	58	-11.56	-5.960	24.77
Dim-E2-061	61	-11.67	-6.546	24.16
Dim-E2-065	64	-11.58	-6.077	24.65
Dim-E2-067	67	-11.24	-6.175	24.54
Dim-E2-070	70	-12.11	-6.531	24.18
Dim-E2-073	73	-11.81	-5.985	24.74
Dim-E2-076	76	-11.68	-6.147	24.57
Dim-E2-079	79	-11.52	-6.209	24.51
Dim-E2-082	82	-11.72	-6.151	24.57
Dim-E2-085	85	-11.76	-5.747	24.99
Dim-E2-088	88	-11.30	-5.825	24.91
Dim-E2-091	91	-11.04	-6.166	24.55
Dim-E2-094	94	-10.92	-5.921	24.81
Dim-E2-097	97	-11.51	-5.971	24.76
Dim-E2-100	100	-12.07	-6.431	24.28
Dim-E2-103	103	-11.64	-6.038	24.69
Dim-E2-106	106	-12.10	-6.387	24.33
Dim-E2-109	109	-11.47	-6.210	24.51
Dim-E2-112	112	-11.46	-5.954	24.77
Dim-E2-115	115	-11.19	-6.129	24.59
Dim-E2-118	118	-11.16	-5.616	25.12
Dim-E2-121	121	-11.11	-5.988	24.74
Dim-E2-124	124	-11.08	-5.810	24.92
Dim-E2-127	127	-10.98	-5.856	24.87
Dim-E2-130	130	-11.38	-5.670	25.07
Dim-E2-133	133	-11.14	-5.963	24.76

Dim-E2-136	136	-11.12	-5.710	25.02
Dim-E2-139	139	-10.39	-5.877	24.85
Dim-E2-142	142	-10.83	-5.881	24.85
Dim-E2-145	145	-11.46	-5.893	24.83
Dim-E2-148	148	-11.60	-5.918	24.81
Dim-E2-151	151	-12.11	-5.912	24.82
Dim-E2-154	154	-12.55	-5.842	24.89
Dim-E2-157	157	-12.54	-6.099	24.62
Dim-E2-160	160	-12.08	-5.760	24.97
Dim-E2-163	163	-12.03	-6.189	24.53
Dim-E2-166	166	-11.46	-5.776	24.96
Dim-E2-169	169	-11.24	-5.553	25.19
Dim-E2-175	175	-11.70	-5.733	25.00
Dim-E2-178	178	-11.50	-5.755	24.98
Dim-E2-181	181	-11.39	-6.024	24.70
Dim-E2-184	184	-11.40	-5.923	24.80
Dim-E2-187	187	-10.62	-5.900	24.83
Dim-E2-190	190	-10.89	-5.827	24.90
Dim-E2-193	193	-11.26	-6.149	24.57
Dim-E2-196	196	-10.78	-5.840	24.89
Dim-E2-199	199	-11.44	-6.053	24.67
Dim-E2-202	202	-11.48	-5.995	24.73
Dim-E2-205	205	-10.66	-5.835	24.89
Dim-E2-208	208	-11.06	-5.989	24.74
Dim-E2-211	211	-10.71	-5.911	24.82
Dim-E2-214	214	-10.10	-5.739	24.99
Dim-E2-217	217	-9.943	-5.846	24.88
Dim-E2-220	220	-9.495	-5.365	25.38
Dim-E2-223	223	-8.948	-4.893	25.87
Dim-E2-226	226	-8.687	-5.040	25.71
Dim-E2-229	229	-8.901	-5.116	25.64
Dim-E2-232	232	-9.156	-5.308	25.44
Dim-E2-235	235	-9.776	-5.613	25.12
Dim-E2-238	238	-9.172	-5.400	25.34
Dim-E2-241	241	-8.645	-5.115	25.64
Dim-E2-244	244	-8.223	-5.358	25.39
Dim-E2-247	247	-7.972	-5.239	25.51
Dim-E2-250	250	-8.774	-5.338	25.41
Dim-E2-253	253	-8.814	-5.555	25.18
Dim-E2-256	256	-9.074	-5.619	25.12
Dim-E2-259	259	-8.583	-5.203	25.55
Dim-E2-264	264	-7.652	-5.189	25.56
Dim-E2-270	270	-8.63	-5.593	25.14
Dim-E2-276	276	-7.771	-5.243	25.51
Dim-E2-282	282	-8.951	-5.783	24.95
Dim-E2-288	288	-8.055	-5.577	25.16
Dim-E4-0035	3.5	-8.649	-5.073	25.68
Dim-E4-0115	11.5	-9.06	-5.406	25.34
Dim-E4-0180	18	-8.576	-5.332	25.41
Dim-E4-0245	24.5	-9.305	-5.569	25.17
Dim-E4-0300	30	-9.18	-4.956	25.80
Dim-E4-0370	37	-9.018	-5.551	25.19
Dim-E4-0415	41.5	-8.855	-5.582	25.16
Dim-E4-0485	48.5	-9.053	-5.580	25.16
Dim-E4-0540	54	-8.812	-5.386	25.36
Dim-E4-0615	61.5	-9.445	-5.671	25.06
Dim-E4-0695	69.5	-9.554	-5.717	25.02
Dim-E4-0780	78	-9.188	-5.505	25.24
Dim-E4-0945	94.5	-8.439	-5.163	25.59
Dim-E4-1000	100	-8.905	-5.437	25.31
Dim-E4-1080	108	-9.129	-5.512	25.23

Dim-E4-1155	115.5	-9.21	-5.487	25.25
Dim-E4-1235	123.5	-9.242	-5.426	25.32
Dim-E4-1310	131	-9.414	-5.433	25.31
Dim-E4-1370	137	-8.88	-5.399	25.34
Dim-E4-1455	145.5	-8.08	-5.271	25.48
Dim-E4-1535	153.5	-7.586	-5.112	25.64
Dim-E4-1600	160	-7.019	-5.186	25.56
Dim-E4-1655	165.5	-7.665	-5.193	25.56
Dim-E4-1760	176	-7.315	-5.020	25.74
Dim-E4-1830	183	-7.607	-5.069	25.68
Dim-E4-1920	192	-7.486	-5.001	25.75
Dim-E4-1985	198.5	-7.88	-5.369	25.38
Dim-E4-2030	203	-8.413	-5.356	25.39
Dim-E4-2110	211	-8.262	-5.287	25.46
Dim-E4-2195	219.5	-8.321	-5.373	25.37
Dim-E4-2275	227.5	-8.245	-5.505	25.24
Dim-E4-2360	236	-8.953	-5.638	25.10
Dim-E4-2430	243	-7.862	-5.575	25.16
Dim-E4-2510	251	-9.094	-5.763	24.97
Dim-E4-2605	260.5	-8.954	-5.725	25.01
Dim-E4-2665	266.5	-8.578	-5.525	25.22
Dim-E4-2740	274	-7.664	-5.279	25.47
Dim-E4-2905	290.5	-6.89	-5.344	25.40
Dim-E4-2955	295.5	-7.964	-5.567	25.17
Dim-E4-3035	303.5	-8.398	-6.110	24.61
Dim-E4-3125	312.5	-7.572	-5.677	25.06
Dim-E4-3200	320	-7.449	-5.728	25.01
Dim-E4-3255	325.5	-7.771	-5.761	24.97
Dim-E4-3335	333.5	-8.079	-5.659	25.08
Dim-E4-3425	342.5	-8.353	-5.881	24.85
Dim-E4-3475	347.5	-8.66	-5.791	24.94
Dim-E4-3525	352.5	-7.877	-5.706	25.03
Dim-E4-3580	358	-8.135	-5.660	25.08
Dim-E4-3670	367	-8.558	-5.883	24.85
Dim-E4-3770	377	-7.913	-5.905	24.82
Dim-HR*	Host rock	5.439	-0.714	30.17

*Dim-HR denotes for the dolomitic limestone host-rock for the Dim Cave.

Table S4 Hendy test $\delta^{18}O$ and $\delta^{13}C$ isotope values of the speleothems from the Dim Cave (Alanya, SW Turkey) (values are in per mil: ‰)

Growth period	Sample ID	Depth (mm)	Distance to axes (mm)	$\delta^{13}C$ (VPDB)	$\delta^{18}O$ (VPDB)	$\delta^{18}O$ VSMOW
1st growth phase (~80–90 ka)	H-Dim-E3-261-1	261.0	4.0	-7.062	-5.376	25.37
	H-Dim-E3-261-2	261.0	18.0	-7.745	-6.443	24.27
	H-Dim-E3-261-3	261.0	23.0	-6.834	-5.356	25.39
	H-Dim-E3-261-4	261.0	29.0	-6.442	-5.039	25.72
	H-Dim-E3-261-5	261.0	34.0	-8.078	-5.259	25.49
2nd growth phase (~62–72 ka)	H-Dim-E3-194-1	194.0	9.0	-9.115	-4.589	26.18
	H-Dim-E3-194-2	194.0	17.0	-8.914	-4.591	26.18
	H-Dim-E3-194-3	194.0	23.0	-9.138	-5.102	25.65
	H-Dim-E3-194-4	194.0	30.0	-8.875	-4.543	26.23
	H-Dim-E3-194-5	194.0	36.0	-8.614	-4.392	26.38
3rd growth phase (~40–52 ka)	H-Dim-E3-085-1	8.5	4.0	-7.084	-4.663	26.10
	H-Dim-E3-085-2	8.5	11.0	-7.174	-4.614	26.15
	H-Dim-E3-085-3	8.5	18.0	-6.434	-4.515	26.25
	H-Dim-E3-085-4	8.5	27.0	-7.468	-4.607	26.16
	H-Dim-E3-085-5	8.5	34.0	-7.266	-4.561	26.21
4th growth phase (~13–40 ka)	H-Dim-E3-040-1	4.0	6.0	-5.721	-4.719	26.05
	H-Dim-E3-040-2	4.0	14.0	-5.257	-4.842	25.92
	H-Dim-E3-040-3	4.0	21.0	-5.186	-4.818	25.94
	H-Dim-E3-040-4	4.0	28.0	-5.081	-4.695	26.07
	H-Dim-E3-040-5	4.0	34.0	-5.079	-4.471	26.30
Growth period (10.6–11.9ka)	H-Dim-E2-130-1	130.0	5.0	-11.096	-5.633	25.10
	H-Dim-E2-130-2	130.0	13.0	-11.299	-5.589	25.15
	H-Dim-E2-130-3	130.0	20.0	-11.036	-5.704	25.03
	H-Dim-E2-130-4	130.0	27.0	-10.704	-5.449	25.29
	H-Dim-E2-130-5	130.0	34.0	-10.498	-5.310	25.44
Growth period (12.8–12.9 ka)	H-Dim-E4-178-1	178.0	5.5	-7.393	-5.637	25.10
	H-Dim-E4-178-2	178.0	11.0	-7.346	-4.995	25.76
	H-Dim-E4-178-3	178.0	16.0	-7.367	-4.996	25.76
	H-Dim-E4-178-4	178.0	23.0	-7.531	-4.967	25.79
	H-Dim-E4-178-5	178.0	29.0	-7.651	-5.125	25.63

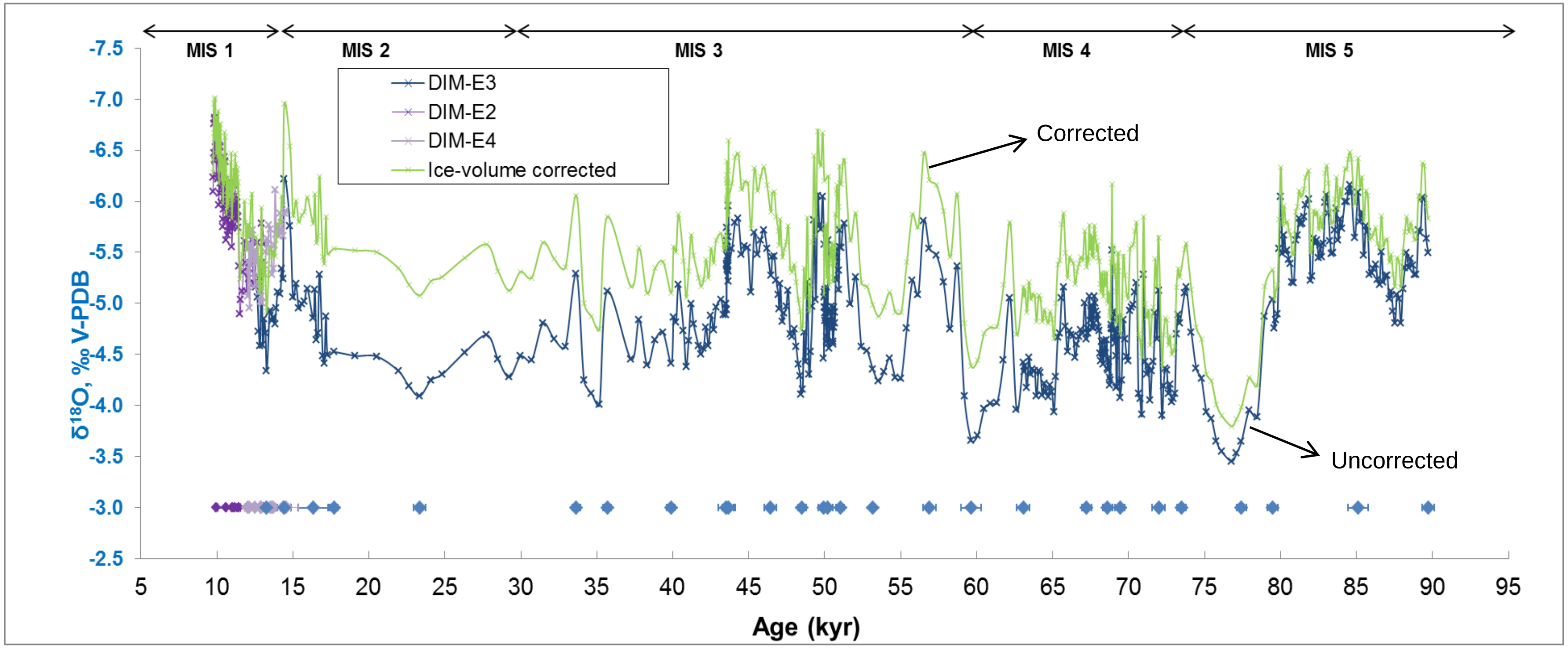

Figure S6 Ice volume-corrected stalagmite $\delta^{18}O$ record of the Dim Cave. Light green curve shows the composite stalagmite $\delta^{18}O$ record after correction for ice volume-related $\delta^{18}O$ contributions to seawater[4]. Blue, purple and light purple curves represent uncorrected $\delta^{18}O$ records of stalagmites Dim-E3, Dim-E2, and Dim-E4, respectively. Corresponding-coloured diamonds show U-series ages of the stalagmites. Age models are constructed by linear interpolation.

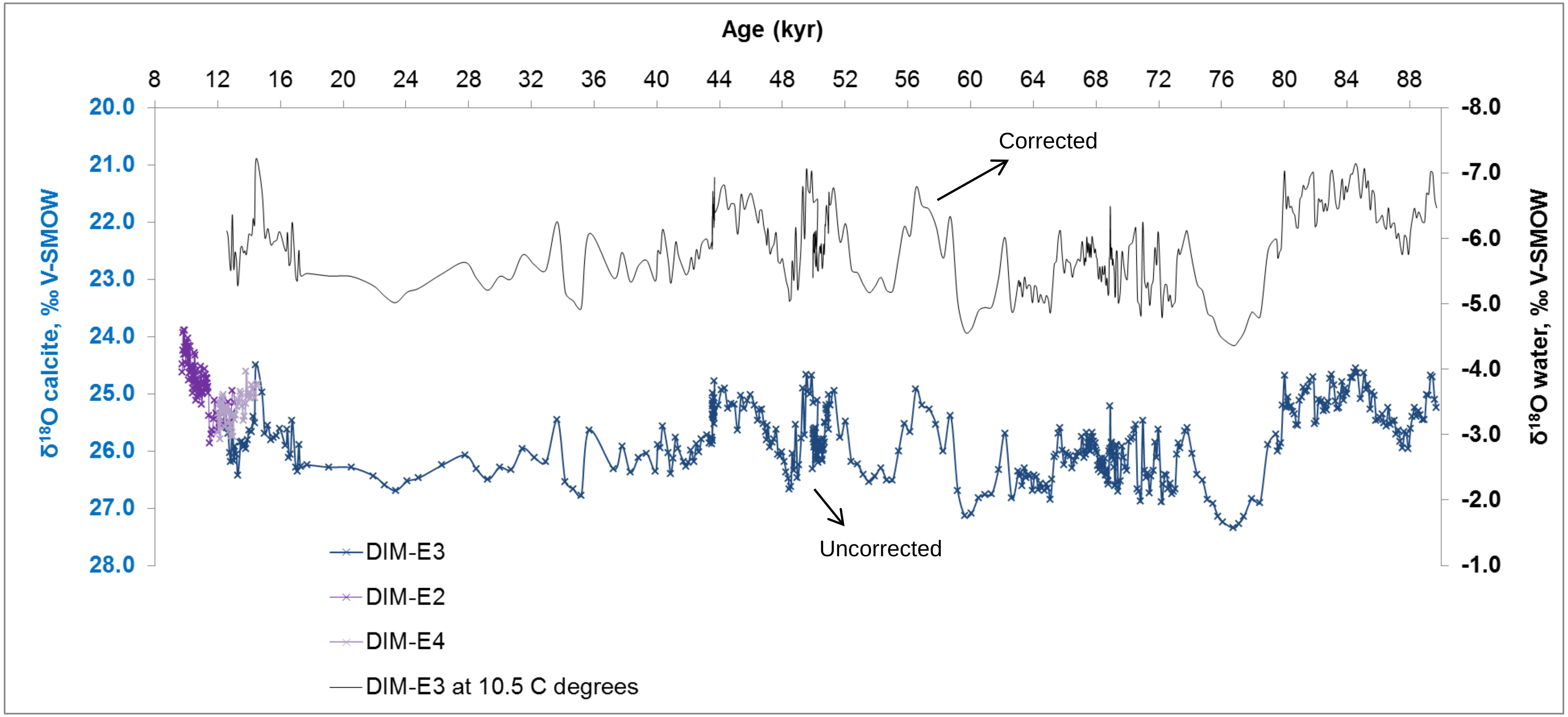

Figure S7 Calcite-water fractionation-corrected stalagmite $\delta^{18}O$ record of the Dim Cave. Light black curve shows composite $\delta^{18}O_{water}$ record after applying calcite-water fractionation equation[5] on $\delta^{18}O_{calcite}$, assuming 10.5 °C (8 degrees lower than today[1,6,7]) within the cave during glacial times. Blue, purple and light purple curves represent uncorrected $\delta^{18}O$ records of Dim-E3, Dim-E2, and Dim-E4, respectively. Note that correction is applied only to Dim-E3, which formed between ~90 and 13 kyr during the last glaciation.

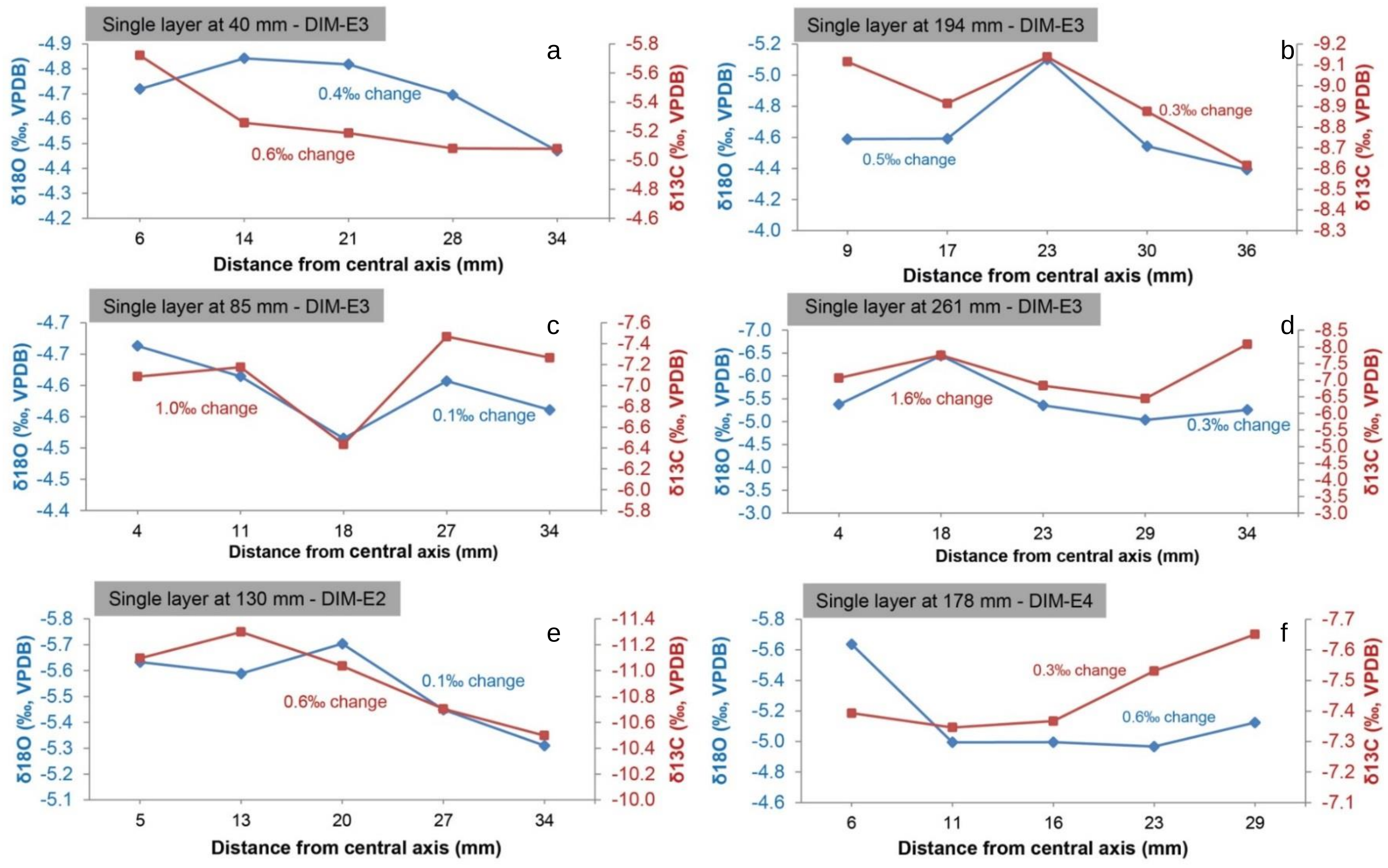

Figure S8 $\delta^{18}O$ and $\delta^{13}C$ plots for Hendy tests[8] along individual layers of Dim-E3 (a, b, c, and d), Dim-E2 (e) and Dim-E4 (f). Isotopic values are plotted as distances away from the central growth axis. Note that the $\delta^{18}O$ values do not vary by more than 0.5‰ along the majority of tested growth layers (except for Dim-E4; f) and do not correlate with $\delta^{13}C$ values (R^2= 0.2), suggesting that the stalagmites were deposited at or close to quasi-isotopic equilibrium.

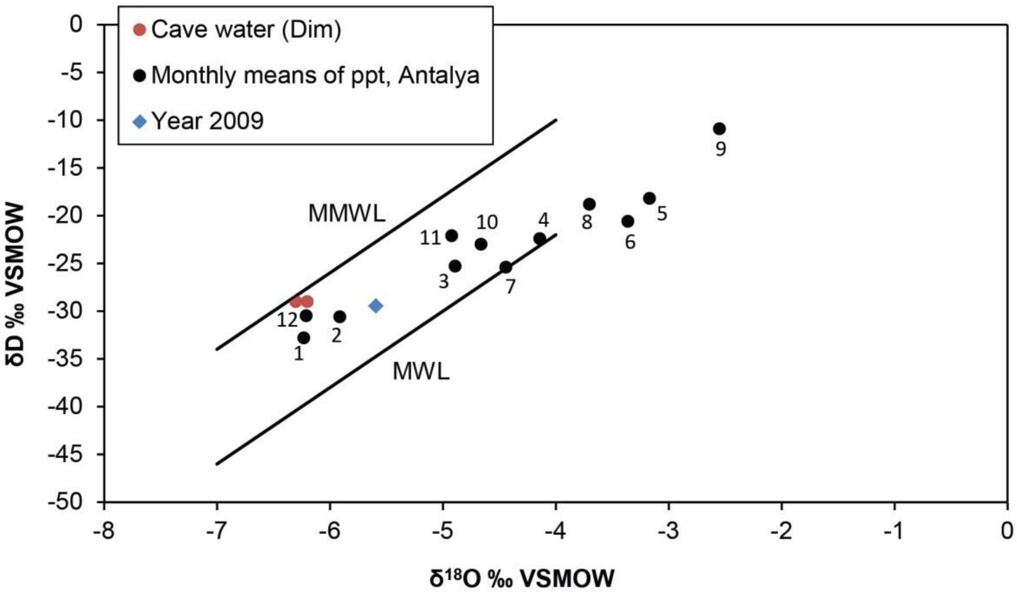

Figure S9 $\delta^{18}O$ and δD relationships of rainfall for the GNIP Antalya station for the period 1963–2009[3]; numbers indicate the respective month in Northern Hemisphere. Modern cave water values are very close to winter rainfall and most recent 2009 rainfall values. The Mediterranean Meteoric Water Line (MMWL)[9] and Meteoric Water Line (MWL)[10] are shown as references. Note that the winter rainfall data plot close to MMWL, while summer/spring rainfall show global MML signals.

Alternative Hypotheses for the interpretation of Dim isotope record

1. Temperature-based interpretation

The temperature dependence of speleothem $\delta^{18}O$ is variable and closely linked to the site conditions[11]. In principle the regional mean annual temperature over the cave is assumed to control the $\delta^{18}O$ of calcite deposited from drip waters. Thus, $\delta^{18}O$ of calcite will reflect more depleted values during glacial periods and vice versa for the interglacials (e.g. Holocene), which is opposite to what is observed in the Dim Cave. The Dim record shows isotopically lighter values (from -4.5 to -7‰ VPDB; Fig. 2c) in the Holocene than in the glacial periods[6,12]. Temperature dependence of isotopic signatures of speleothems are well-exemplified from the Sofular record from Black Sea region[13] where $\delta^{18}O$ values of calcite becomes less negative towards Holocene (~-13 to -8‰ VPDB; Fig. 2a).In contrast, the Dim record cannot reflect temperature changes. This situation is quite common in the literature for relatively close sites. For example some records from New Zealand follow temperature[14] while others sites have a reverse isotopic signal from the $\delta^{18}O$[15].

2. Volume of rainfall (amount effect)

The amount effect is widely interpreted as a control on the isotopic composition of speleothems. In the case of the last glaciation from DIM, this interpretation is difficult to sustain. We have independent evidence from two sources that show that conditions during glacial periods are drier or at least no wetter than in the Holocene. The PMIP reconstruction for the LGM[16] suggests rainfall values that are virtually identical to modern. More tellingly, our speleothem growth rates from Dim-E3 (Fig. S2a) reveal that growth rates are positive during glacial times but are much slower than the early Holocene (which is known to be wet in the EM[17,18]). The fact that the moisture balance is positive in glacial times does not imply higher rainfall. In fact, reduced evaporative flux due to depressed temperatures, reduced forest cover and increased snow-cover all divert the limited moisture flow to groundwater pathways. It is highly likely that all of the glacial period has less rainfall than the Holocene.

3. Seasonality of Rainfall

SW Turkey is located in the heart of the westerly flow zone. At the present day almost no precipitation occurs in summer and if the shoulder months are taken into account winter rainfall dominates completely[3]. This is evident from all the modern climatological data[19–21]. For the last glacial cycle, the coldest period is the LGM and the PMIP models clearly demonstrate that there is no switch from winter to summer dominated rainfall in this part of the EM[16]. It is virtually impossible for monsoonal rain to penetrate this far north under modern conditions and the likelihood under glacial conditions is significantly reduced.

4. Evaporation effects

Evaporation effects on the speleothem isotopic signal are important. This is actually embedded in our interpretation because the air mass changes involve a complex mix of changed temperature and evaporation due to source area, cloud cover and other variables.

5. Sea air temperature

The changes in ocean source moisture will relate to a series of independent variables. These include; 1) regional changes in air temperature, which is also caused by the air masses passing over the region; 2) changes in the thermohaline circulation in the eastern Mediterranean[22]. In theory this is hard to incorporate, but the thermohaline circulation reflects primarily wind stress across the basin and the through flow to/from the Atlantic. Both of these are directly related to the westerly circulation and what is happening in the North Atlantic source area, and so are closely linked with the westerly air masses. We also note that the smaller scale changes in

deep/intermediate water formation relate to decadal scale changes in the Mediterranean Basin[23] and these are likely to be integrated out of a millennial scale record.

Supplementary References

1. Gündalı, N., Nazik, L., Soylu, C. ., Aksoy, B. & Beydeş, S. *Dim Mağarası (Alanya) Araştırma Raporu, Mimari ve Elektrikasyon Uygulama Projeleri (in Turkish, unpublished report no. 369)* (1989).

2. Frisia, S. Ã. Microstratigraphic logging of calcite fabrics in speleothems as tool for palaeoclimate studies. *Int. J. Speleol.* **44,** 1–16 (2015).

3. IAEA/WMO. Global Network of Isotopes in Precipitation. The GNIP Database. (2015). Accessible at < http://www.iaea.org/water>

4. Bintanja, R. & van de Wal, R. S. W. North American ice-sheet dynamics and the onset of 100,000-year glacial cycles. *Nature* **454,** 869–72 (2008).

5. Kim, S.-T. & O'Neil, J. R. Equilibrium and nonequilibrium oxygen isotope effects in synthetic carbonates. *Geochim. Cosmochim. Acta* **61,** 3461–3475 (1997).

6. Hughes, P. D., Gibbard, P. L. & Ehlers, J. Timing of glaciation during the last glacial cycle: evaluating the concept of a global "Last Glacial Maximum" (LGM). *Earth-Science Rev.* **125,** 171–198 (2013).

7. Sarıkaya, M. A., Çiner, A., Haybat, H. & Zreda, M. An early advance of glaciers on Mount Akdağ, SW Turkey, before the global Last Glacial Maximum; insights from cosmogenic nuclides and glacier modeling. *Quat. Sci. Rev.* **88,** 96–109 (2014).

8. Hendy, C. H. The isotopic geochemistry of speleothems-I. The calculation of the effects of different modes of formation on the isotopic composition of speleothems and their applicability as paleoclimatic indicators. *Geochim. Cosmochim. Acta* **35,** 80l–824 (1971).

9. Gat, J. R. & Carmi, I. Effect of climate changes on the precipitation patterns and isotopic composition of water in a climate transition zone: Case of the Eastern Mediterranean Sea area. in *Influ. Clim. Chnage Clim. Var. Hydrolic Regime Water Resour.* **1968,** 513–524 (IAHS Publ. no. 168, 1987).

10. Craig, H. Isotopic Variations in Meteoric Waters. *Science* **133,** 1702–1703 (1961).

11. McDermott, F. Palaeo-climate reconstruction from stable isotope variations in speleothems: a review. *Quat. Sci. Rev.* **23,** 901–918 (2004).

12. Essallami, L., Sicre, M. a., Kallel, N., Labeyrie, L. & Siani, G. Hydrological changes in the Mediterranean Sea over the last 30,000 years. *Geochemistry, Geophys. Geosystems* **8,** 1–11 (2007).

13. Fleitmann, D. *et al.* Timing and climatic impact of Greenland interstadials recorded in stalagmites from northern Turkey. *Geophys. Res. Lett.* **36,** 1–5 (2009).

14. Williams, P. W., King, D. N. T., Zhao, J.-X. & Collerson, K. D. Late Pleistocene to Holocene composite speleothem 18O and 13C chronologies from South Island, New Zealand—did a global Younger Dryas really exist? *Earth Planet. Sci. Lett.* **230,** 301–317 (2005).

15. Hellstrom, J., Mcculloch, M. & Stone, J. A Detailed 31 , 000-Year Record of Climate and Vegetation Change , from the Isotope Geochemistry of Two New Zealand Speleothems. *Quat. Res.* **178,** 167–178 (1998).

16. Braconnot, P. *et al.* Results of PMIP2 coupled simulations of the Mid-Holocene and Last Glacial Maximum – Part 1: experiments and large-scale features. *Clim. Past* **3,** 261–277 (2007).

17. Jones, M. D., Roberts, C. N. & Leng, M. J. Quantifying climatic change through the last glacial–interglacial transition based on lake isotope palaeohydrology from central Turkey. *Quat. Res.* **67,** 463–473 (2007).

18. Lisker, S., Vaks, A., Bar-Matthews, M., Porat, R. & Frumkin, A. Late Pleistocene palaeoclimatic and palaeoenvironmental reconstruction of the Dead Sea area (Israel), based on speleothems and cave stromatolites. *Quat. Sci. Rev.* **29,** 1201–1211 (2010).

19. Jex, C. N. *et al.* A 500yr speleothem-derived reconstruction of late autumn–winter precipitation, northeast Turkey. *Quat. Res.* **75,** 399–405 (2011).

20. Sarıkaya, M. A., Zreda, M., Çiner, A. & Zweck, C. Cold and wet Last Glacial Maximum on Mount Sandıras, SW Turkey, inferred from cosmogenic dating and glacier modeling. *Quat. Sci. Rev.* **27,** 769–780 (2008).

21. Türkeş, M. & Erlat, E. Winter mean temperature variability in Turkey associated with the North Atlantic Oscillation. *Meteorol. Atmos. Phys.* **105,** 211–225 (2009).

22. Bar-Matthews, M., Ayalon, A., Kaufman, A. & Wasserburg, G. J. The Eastern Mediterranean paleoclimate as a reflection of regional events: Soreq cave, Israel. *Earth Planet. Sci. Lett.* **166,** 85–95 (1999).

23. Pinardi, N. & Masetti, E. Variability of the large scale general circulation of the Mediterranean Sea from observations and modelling: a review. *Palaeogeogr. Palaeoclimatol. Palaeoecol.* **158,** 153–173 (2000).
